# Graphene Nanostructures by Pulsed Laser Ablation in Liquids: A Review

**DOI:** 10.3390/ma15175925

**Published:** 2022-08-27

**Authors:** Reem M. Altuwirqi

**Affiliations:** Physics Department, Faculty of Science, King Abdulaziz University, P.O. Box 42805, Jeddah 21551, Saudi Arabia; raltuwirqi@kau.edu.sa

**Keywords:** pulsed laser ablation in liquids, PLAL, graphene, graphene quantum dots

## Abstract

High-quality graphene has demonstrated remarkable mechanical, thermal, electronic, and optical properties. These features have paved the road for the introduction of graphene into numerous applications such as optoelectronics and energy devices, photodegradation, bioimaging, photodetectors, sensors, and biosensors. Due to this, graphene research has accelerated exponentially, with the aim of reaching a sustainable large-scale production process of high-quality graphene that can produce graphene-based technologies at an industrial scale. There exist numerous routes for graphene fabrication; however, pulsed laser ablation in liquids (PLAL) has emerged as a simple, fast, green, and environmentally friendly method as it does not require the use of toxic chemicals. Moreover, it does not involve the use of expensive vacuum chambers or clean rooms. However, the great advantage of PLAL is its ability to control the size, shape, and structure of the produced nanostructures through the choice of laser parameters and liquid used. Consequently, this review will focus on recent research on the synthesis of graphene nanosheets and graphene quantum dots via PLAL and the effect of experimental parameters such as laser wavelength, pulse width, pulse energy, repetition rate, irradiation time, and liquid media on the produced nanostructures. Moreover, it will discuss extended PLAL techniques which incorporate other methods into PLAL. Finally, different applications that utilize nanostructures produced by PLAL will be highlighted. We hope that this review will provide a useful guide for researchers to further develop the PLAL technique and the fabrication of graphene-based materials.

## 1. Introduction

### 1.1. Why Graphene?

The element carbon has many allotropes. These include graphite, diamond, fullerene, carbon nanotubes, and graphene, and other forms are predicted [1]. In graphene, carbon atoms are bonded in a honeycomb crystal lattice that is one-atom-thick, forming a 2D structure. The isolation of a single sheet of carbon was discovered by Andre Geim and Konstantin Novoselovan and was an important scientific achievement that was awarded a Noble Prize in 2010 [2]. Since then, many investigations have been conducted to study graphene and all its features that differ greatly from its bulk formation [3].

High-quality graphene demonstrates remarkable properties. These include, but are not limited to, high electron mobility of about 2.5 × 10^5^ cm^2^ V^−1^s^−1^ at room temperature [4], high thermal conductivity reaching 3000 Wm^−1^K^−1^ [5], a high Young’s modulus of about 1 TPa [6], impermeability to liquids and gases [7], high densities of electric current [8], high transparency [9], and the ability to be chemically functionalized [10]. Moreover, one of the dramatic features that has been reported recently is the ability to switch graphene bilayers between being an insulator to superconductor through twisting by the so-called magic-angle [11]. These mechanical, thermal, electronic, and optical features of graphene paved the road for its introduction into numerous applications such as optoelectronics and energy devices [12,13], photodegradation [14], bioimaging [15,16], photodetectors [17], sensors, and biosensors [18].

A major challenge for graphene production is the ability to scale it up for commercial use. This includes the ability to manufacture, in a reproducible manner, large graphene sheets that are defect free and single-crystal. Moreover, the thickness of the graphene sheets can be controlled; the thickness has a direct effect on graphene properties and thus the applications for which it can be used [19]. For example, single-layer graphene exhibits high carrier mobilities [20], bi-layers demonstrate variable band gap [21], and multi-layers provide enhanced ohmic contacts. Another crucial point regarding the commercialization of graphene products is the establishment of standards that identify the quality of the manufactured graphene, such as in the efforts of the Graphene Council [22]. As for the cost of graphene production, there are attempts to reduce the cost of fabrication using plants [15] and biomass [23].

### 1.2. Methods of Synthesizing Graphene (Chemical/Physical)

The production of graphene at a large scale is a very attractive goal. However, the fabrication of well-crystallized carbon nanostructures is considered difficult. This difficulty stems from the requirement of ultrahigh temperatures and pressure to obtain good crystallinity. Hence, conventional methods may not be the best choice since they require high temperature and high vacuum conditions. Moreover, it might be difficult to control the size of the produced nanostructures.

Synthesis of graphene has been accomplished by different routes. Usually, they are characterized by a top-down or a bottom-up approach. Moreover, these methods are categorized as being either through chemical or physical methods. Chemical methods include mechanical exfoliation of graphite, chemical vapor deposition (CVD), and epitaxial growth on a substrate [24,25,26,27]. However, these methods require expensive equipment and their yield is very low for industrial-scale production.

Among the physical methods used to produce graphene from graphite is pulsed laser ablation. Laser ablation (LA) can occur in vacuum, argon, or air [19]. However, there are many advantages when performed in liquids via the one-step pulsed laser ablation in liquid technique (PLAL) [28,29,30]. This method is used to produce graphene through laser irradiation of a graphite target submerged in a liquid medium. The laser–target interaction within the liquid medium provides the high temperature and pressure needed for nanostructure fabrication. Through this one-step method, it was possible to exfoliate graphene in a liquid medium, cause nucleation for graphene quantum dots synthesis, or directly deposit graphene on a foil [31,32]. Not only was this method successful in the production of carbon nanostructures, but it has shown to be effective in the synthesis of metal and metal-oxide nanostructures [33,34,35,36]. The factors controlling the production of nanostructures via PLAL are of interest to many researchers [36,37].

### 1.3. Why PLAL?

The main focus of this review is to highlight the employment of the PLAL method for the fabrication of graphene nanosheets (GNS) and graphene quantum dots (GQDs). This method involves the ablation of a solid graphite target or graphite flakes submerged in a liquid medium by using laser pulses. After production, the produced nanostructures are found in a colloidal solution. It is worth mentioning that laser ablation can also be performed in a gaseous environment; while this is not within the scope of this review, it is covered in other literature [34].

**Figure 1:**
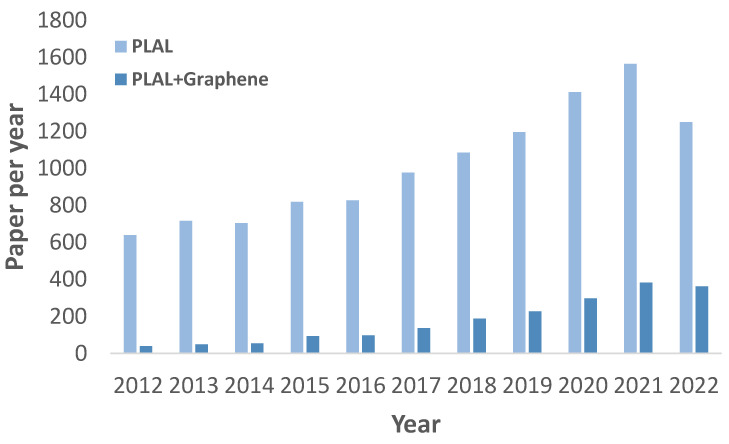
Publications in the field of pulsed laser ablation in liquids (PLAL: general nanostructures, PLAL + Graphene: graphene-based nanostructures via PLAL). Database: Science Direct.

In comparisons to other nanostructure fabrication methods, PLAL is regarded as a straightforward method that is easy and fast. Moreover, PLAL is regarded as a green and environmentally friendly method as it does not require the use of toxic chemicals. The simple experimental setup for this method is also an attractive feature, as it does not require any expensive vacuum chambers, clean rooms, or complicated procedures. It is worth noting that PLAL is termed differently by many researchers; however, they indicate the same technique. Some of these terms are pulsed laser-induced reactive quenching (PLIRQ), laser ablation in a liquid medium (LALM) [29], laser ablation of solids in solution (LASiS) [37], and liquid phase pulsed laser fragmentation (LP-PLF) [38]. PLAL have gained high interest in the past decade for the formation of various nanostructures in general and for graphene-based nanostructures specifically as illustrated in Figure 1.

**Figure 2:**
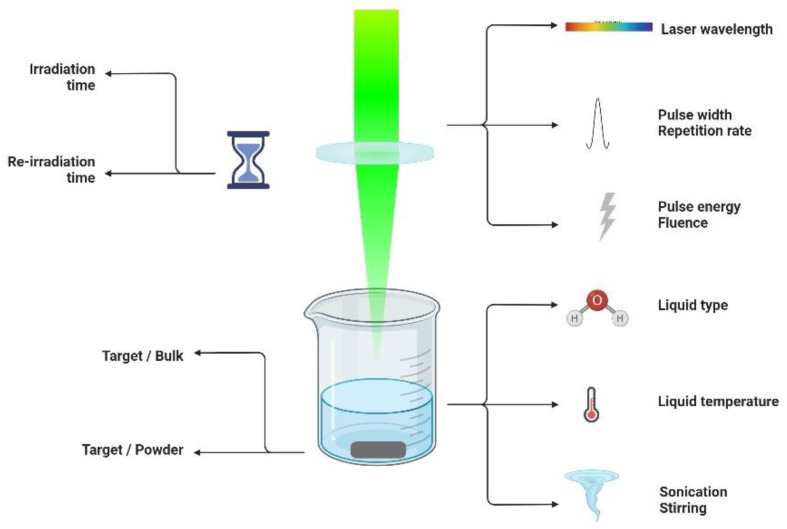
PLAL experimental parameters. (Created with BioRender.com).

One of the great advantages of the PLAL technique is the ability to combine different target materials and liquids in an unlimited combination to produce diverse nanostructures in liquids of choice as can be seen in Section 3. Another advantage of PLAL is the ability to control the size, shape, size distribution, and structure of the produced nanostructures through the choice of laser parameters used during the ablation [37,39]. These parameters include the laser wavelength, pulse width, pulse repetition rate, pulse energy, and fluence (energy per unit area) [37]. Moreover, the irradiation time during ablation, or the re-irradiation after its completion, is a key factor affecting the size of the produced nanostructures [40]. These factors are illustrated in Figure 2.

From the point of view of applications, nanostructures synthesized by utilizing the PLAL technique have high stability and suitability for employment in biological and biochemical applications [16]. This is due to the fact that they can be fabricated using water only, causing them to be ultrapure and free from any contamination [40]. Furthermore, they can be made without capping ligands on their surfaces and can be functionalized at a later stage if required [34].

## 2. Fundamentals of PLAL

### 2.1. Experimental Setup for Nanostructure Formation via PLAL

**Figure 3:**
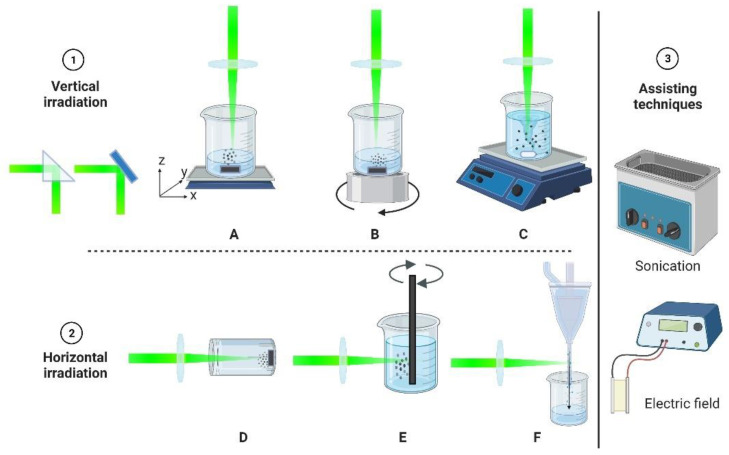
Different configurations for a PLAL setup, (**A**) XYZ translation stage, (**B**) rotational stage, (**C**) magnetic stirring, (**D**) fixed horizontally, (**E**) rotational rod, and (**F**) liquid jet flow. (Created with BioRender.com).

There are various designs to conduct a PLAL experiment. The most common is the vertical configuration where the laser beam irradiates the target vertically, where the laser beam can be redirected using a prism or mirror. The target is placed at the bottom of a container that is filled with a liquid. The container is placed on top of an XYZ translation stage (Figure 3A) or a rotational stage (Figure 3B) to assist in the movement of the target under laser irradiation to avoid crating. When the target is a powder, a magnetic stirrer is used to distribute the particles under the laser beam (Figure 3C). Furthermore, the laser can be unfocused or focused with the aid of a lens. The second group of configurations is the horizontal setup. In this formation, a target could be fixed to the bottom of cuvette that is filled with a liquid and sealed (Figure 3D). The cuvette is then fixed on an XYZ translation stage, and the laser beam irradiates the target horizontally [34]. The advantage of this setup is that it assists in focusing the laser beam on the target surface. Moreover, since the liquid is sealed, there will be no evaporation in the liquid and therefore the focusing conditions do not change as in the vertical irradiation, for which the container is open and the beam might become out of focus with the change in the liquid level. If the target is rod-shaped, then it can be made to rotate around its axis while being dipped in the liquid during the laser exposure, and the laser beam irradiates it horizontally (Figure 3E) [41]. Lastly, the free liquid jet configuration (Figure 3F) has an advantage in its ability to precisely correlate the laser energy and material responses; however, it is rarely used [42,43]. All configurations can be extended by incorporating external methods, such as including sonication or a uniform electric field.

### 2.2. Laser–Matter Interaction

A knowledge of laser–matter interaction is necessary for the understanding of nanostructure formation in a PLAL technique. A laser beam is described by its energy and diameter, among other parameters. When this beam is focused to a smaller area via optical components, such as a lens, all the energy of the beam is focused into an area smaller than the original diameter of the laser beam [44]. Therefore, a more suitable description of a laser energy in laser–matter interaction is fluence. Fluence is defined as the energy per unit area (J/cm^2^). Each material has a specific value of what is known as ablation threshold. The material ablation threshold is determined by the minimum fluence that causes the removal of the material. When the laser fluence is greater than the material’s threshold, the material will be ablated [34].

**Figure 4:**
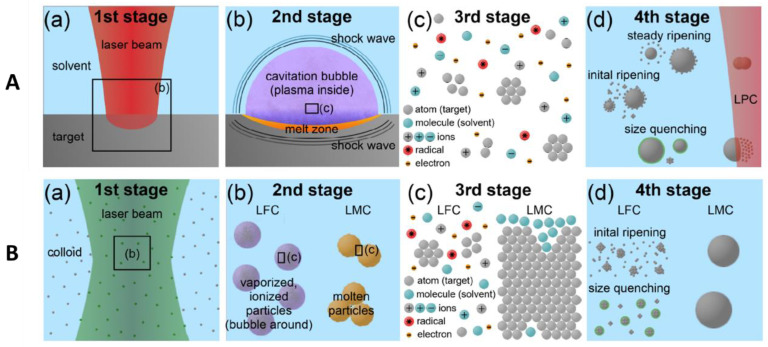
Laser–matter interaction stages for (**A**) laser synthesis of colloids (LSC): (**a**) laser irradiation of target, (**b**) material ablation and the formation of plasma, cavitation bubble and shock wave, (**c**) nanostructure formation, and (**d**) ripening, quenching and re-irradiation of nanostructures. (**B**) laser processing of colloids (LPC): (**a**) laser irradiation of colloidal particles, (**b**) material phase changes, (**c**) particles formation, and (**d**) ripening and quenching of produced particles [45].

Laser synthesis of colloids (LSC) to generate nanoparticles in a PLAL process can be classified into four stages [45]. First, the laser beam irradiates the target while immersed in a liquid solution. Second, ablation takes place and a plasma plume is generated whether the medium is air or liquid. The plasma plume then expands against the surrounding environment. In the case of ablation in a liquid medium, the expanding plasma plume experiences a stronger confinement. This results in the plume having higher temperature, pressure, and density. In turn, the surrounding liquid is also heated to the same high temperature, causing it to vaporize and form what is known as a liquid-plasma. Hence, in this stage, a plasma plume develops a cavitation bubble and two shock waves. An extensive study of the cavitation dynamics and shockwaves has been undertaken [46,47,48,49]. When the two plasmas mix together, a chemical reaction can take place between the species of the plume and the liquid-plume, resulting in the formation of the nanoparticles. This comprises the third stage. In the final fourth stage, nanoparticles may undergo ripening where their size increases, quenching where their size decreases, or be re-irradiated by the laser beam and be further processed (laser processing of colloids (LPC)) (Figure 4A) [45].

An LPC process occurs when the target is larger in size compared to the size of the laser spot. When the laser spot is larger than the target, as in the case when the target is not a bulk material but colloidal particles, the LPC process can be further categorized into four stages (Figure 4B) [45]. The first stage involves the absorption of the laser photons by the dispersed particles. In the second stage, laser fragmentation of colloids (LFC) and laser melting of colloids (LMC) take place, encompassing processes such as plasma, bubbles, and shockwave formation. At stage three, the LFC contributes to the reduction of the particle size, while the LMC process results in the merging of the particles to form larger spherical particles. The LMC process stops at stage three; however, the LFC process continues into stage four to contribute to the ripening and quenching of the particles’ size [45].

**Figure 5:**
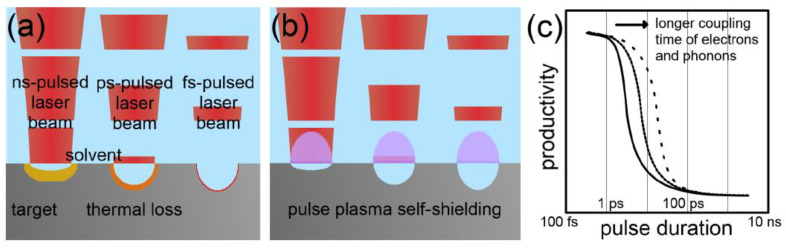
The effect of pulse duration on the laser ablation of a target in liquid (**a**) thermal loss, (**b**) pulse plasma self-shielding, and (**c**) NP productivity vs. pulse duration. [45].

One of the main factors that has a great effect in laser–matter interaction is the temporal width of the laser pulse. Nano-, pico-, and femtosecond lasers interact differently with materials [50]. For example, the laser energy in a long nanosecond pulse causes the material to be ablated through melting and evaporation, therefore transforming the material from solid to liquid to vapor. On the other hand, short pulses with femtosecond duration ablate the material via sublimation, hence transforming the material from solid to vapor directly. Picosecond pulses are an intermediate case between nano- and femtosecond pulses. These are regarded as a general division between the ablation mechanisms in a material, although a more detailed examination will include the electron thermal diffusion length. For instance, a femtosecond laser pulse of high fluence can result in a high heat penetration depth, leading to increased thermal diffusion. In this case, the material will be ablated by melting and vaporization, even though the laser pulse is very short. Furthermore, it was found that the ablation rate is affected by the pulse duration, where longer pulse duration reduces the ablation rate due to heat loss in the target and laser-induced shielding [51]. An illustration of the laser ablation of a target and pulse plasma self-shielding for different pulse durations is shown in Figure 5 [45].

**Figure 6:**
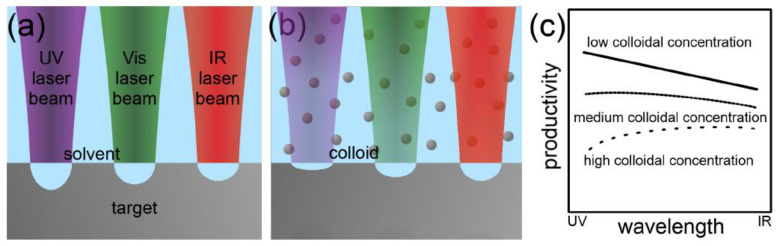
Effect of laser wavelength on the ablation rate in the (**a**) absence and (**b**) presence of colloidal particles, and (**c**) NPs productivity vs. wavelength at different colloidal concentrations [45].

**Figure 7:**
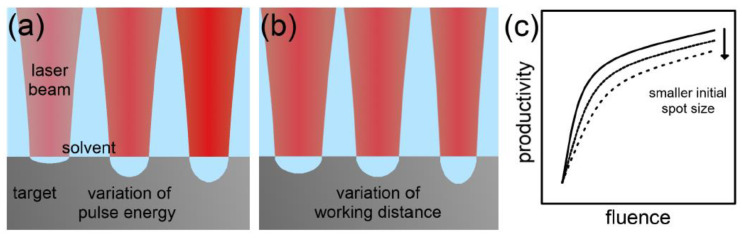
Effect of laser fluence on the ablation rate due to (**a**) pulse energy and (**b**) working distance. (**c**) Productivity of NPs vs. fluence by different initial spot sizes [45].

Other factors that affect the laser–matter interaction are the laser wavelength and fluence. The ablation rate is higher when shorter wavelengths are used in a PLAL process [52]. However, when high colloidal concentrations are present in the solvent, the dispersed particles absorb shorter wavelength photons more than higher wavelength photons, resulting in a reduction in the ablation rate (Figure 6) [45]. As for the laser fluence, the ablation rate is found to grow logarithmically with increasing laser fluence (Figure 7) [45].

A major concern in nanostructure synthesis in general is the ability to control size and size distribution. In PLAL, this is accomplished by controlling the laser and experimental parameters. Factors such as laser wavelength, pulse duration, laser fluence, and repetition rate are linked to the laser instruments. However, more parameters are involved and can control the size of the produced nanostructures. For example, the focal length of the lens used to focus the laser beam, the liquid medium used, its temperature, height above the target, and the target itself and whether it is bulk or flakes. Therefore, regardless of the simplicity of the setup, its factors and their interdependency need to be optimized to develop a protocol for controlled synthesis [53]. Extending the PLAL process by incorporating other techniques, such as the addition of electrical fields [54], sonication [55], or flow of the liquid medium [43], would require the consideration of other parameters that affects the size of the produced nanostructures.

### 2.3. Graphene Formation Mechanisms

Graphite consists of a planar layered structure. Graphene is an individual layer of graphite. Weak Van der Waals bonds are responsible for the bonding between the graphene layers. Due to this, the graphite layers can be easily separated when sufficient energy is provided. The laser energy in the PLAL process can provide such energy to break the weak Van der Waals bonds between the graphite planes to produced graphene nanosheets [24,56].

**Figure 8:**
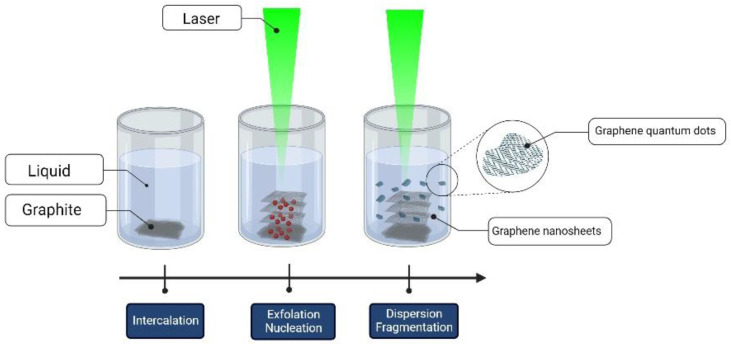
Graphene formation mechanism (Created with BioRender.com).

In PLAL, the energy transferred from the laser to the material is used to perform different processes. Part of the energy is consumed in melting, evaporating, and ionizing the target material to form the nanoparticles. The other part of the energy can be used to break the Van der Waals bonds [57]. The synthesis of nanoparticles via PLAL is typically a bottom-up approach. That is, after melting, evaporation, and ionization, atoms nucleate to form the nanoparticles. However, PLAL can perform laser exfoliation (LE), which is regarded as a top-down approach. When a laser is used to irradiate a graphite target, both bottom-up and top-down process can occur. Single carbon atoms can be detached and then aggregate to form carbon nanoparticles. Moreover, the laser–carbon interaction in the liquid media can thermalize the system, preventing the carbon from reaching the melting temperature. In this case, a single or multi-layer of graphene can be exfoliated when the Van der Waals bonds are broken [58]. Both carbon nanoparticles and graphene nanosheets will be dispersed in the colloidal solution (Figure 8) [58]. An extensive review on liquid-phase exfoliation (LPE) for the formation of graphene nanosheets (GNS) from graphite precursors in liquid media has been undertaken [31,59,60,61].

Although laser energy in the PLAL process can be utilized to produce graphene nanosheets, the choice of liquid media is of high importance. Since graphene is hydrophobic, it tends to agglomerate irreversibly and undergo restacking to form graphite. Hence, the advantages of single- or multi-layer graphene are lost. Therefore, different liquid media that would hinder the stacking of the graphene nanosheets were investigated [31,62].

**Figure 9:**
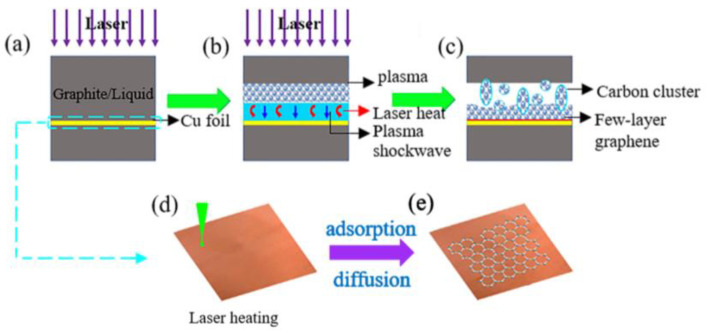
Mechanism of film deposition by PLAL. (**a**) Schematic diagram of laser irradiation of graphite suspension in liquid, (**b**) plasma and shockwave formation, (**c**) production of FLG on Cu substrate, (**d**) fabrication of graphene on Cu substrate, and (**e**) final product after adsorption and diffusion [32].

PLAL has also been used to directly deposit layers of graphene on the surface of a foil [32]. Figure 9 illustrates the process of the deposition. The mechanism of graphene growth on a Cu foil was described [32]. Starting from the law of conservation of energy, the total energy is the summation of the energy reflected, absorbed, and transmitted. Graphite flakes are uniformly suspended in water. Since the absorptivity of graphite is very large, little to none of the laser energy is transmitted to the substrate. The water performs two roles in the process. First, the water acts as an absorbing layer. Second, it acts as a constraining layer. The photothermal process takes place on the interface between the graphite and water. The laser irradiation falling on the material in the water causes small fragmentation of graphene sheets and the ejection of carbon atoms and ions. Furthermore, it causes the ejection of free hydrogen radicals and oxygen ions. This is followed by the formation of dense gas plasma plumes. The expansion of the plasma plume give rise to a high-pressure shock front that reacts physically and chemically with the surrounding liquid environment. The shock front eventually collapses, causing the formation of bubbles. The bubbles expand outwardly at high speed, giving rise to a pressure difference on the sides. As the bubbles contain carbon atoms, carbon ions, and carbon clusters, at the interface with the foil, they exhibit a high-pressure front and the bubbles get deformed and depressed at the interface. This in turn causes a microjet toward the substrate. After cooling, graphene layers are formed on the surface of the Cu foil [32].

### 2.4. Characterizing Graphene Nanostructures

Graphene and graphene-oxide nanostructures have diverse structural and optical properties. To identify these properties, different analytical techniques are employed. These techniques include X-ray diffraction (XRD), X-ray photoelectron spectroscopy (XPS), Raman spectroscopy, UV-Vis absorbance spectroscopy, photoluminescence emission (PL), and Fourier transform infrared spectroscopy (FT-IR). Below, a brief description of these techniques is given with the main features of graphene and graphene-oxide nanostructures yielded by these methods. A summary of these features is listed in Table 1.

#### 2.4.1. XRD Analysis

**Figure 10:**
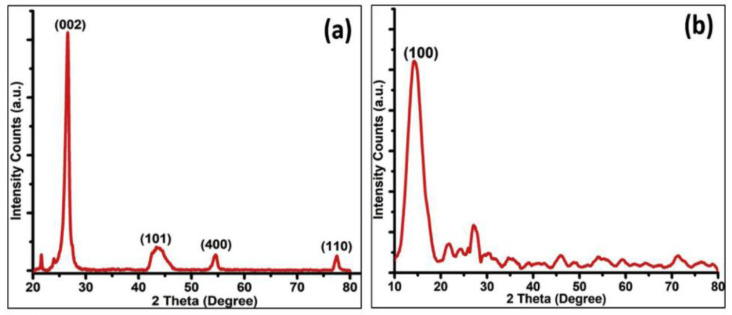
XRD spectrum for (**a**) graphene and (**b**) graphene oxide. Reprinted with permission from Ref. [63]. 2019, Elsevier.

X-ray diffraction (XRD) is an analytical technique that is used in the identification of crystalline materials and provides knowledge of their phase. Furthermore, it can give information on the material’s unit cell dimensions. Graphene is identified by five major peaks in an XRD spectrum. These peaks occur at 26.5°, 42.3°, 44.5°, 54.6°, and 77.4°, which are related, respectively, to the hexagonal planes (002), (100), (101), (004), and (110) of graphitic carbon [63] (Figure 10a). The XRD spectrum for graphene oxide is characterized by a peak positioned at 2θ ≅ 9–11°, which corresponds to the (001) plane. This peak has been reported to shift to 2θ ≅ 12–17° (Figure 10B) depending on the amount of water trapped between adjacent layers of graphene [16,24]. As for the GQDs and GOQDs, it has been reported that they exhibit different structures, such as those of amorphous carbon, diamond-like crystal structure, and graphite or graphite oxide structures [13].

#### 2.4.2. XPS Spectroscopy

XPS is concerned with the analysis of the surface chemistry of a material. XPS analysis provides information on the elemental composition of the material under investigation as well as its chemical and electronic state of the atoms within it.

**Figure 11:**
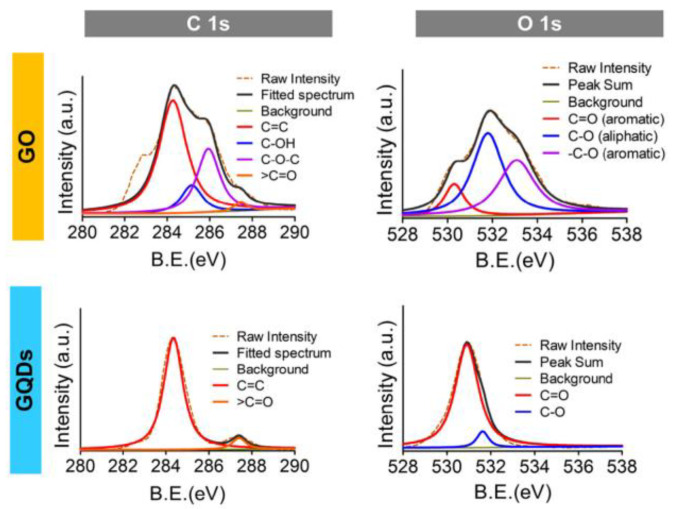
XPS analysis of GO and GOQDs. Reprinted with permission from Ref. [64]. 2019, Elsevier.

When analyzing carbon nanostructures through XPS, two major elements are revealed: carbon and oxygen. These are indicated by two peaks positioned at 284 eV and 532 eV, which correspond to the singlet C 1 s and O 1 s peaks, respectively (Figure 11). By deconvoluting the peaks, it is possible to determine the position of the encompassed bands, indicating the hybridization and functional groups attached to the basal plane or edges of the nanostructures [64].

#### 2.4.3. Raman Spectroscopy

**Figure 12:**
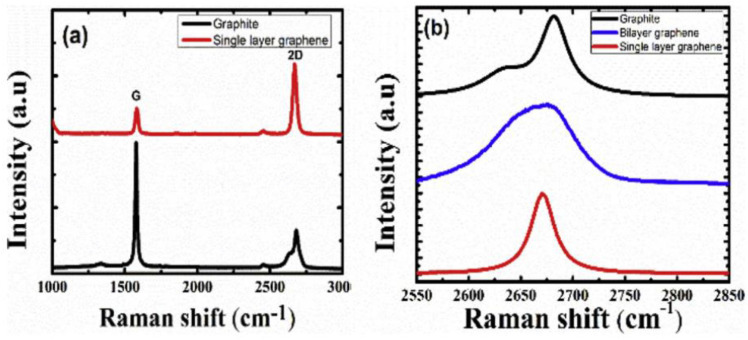
(**a**) Raman spectra for graphite and single-layer graphene and (**b**) 2D band enlarged. Reprinted with permission from Ref. [66]. 2020, Elsevier.

Raman spectroscopy is an analytical technique that measures the vibrational energy modes of a substance and provides both chemical and structural information that assists in identifying the substance through its Raman ‘fingerprint’. Carbon allotropes exhibit distinct bands in a Raman spectrum. The major three bands are the D-band (1330–1360 cm^−1^), G-band (1560–1600 cm^−1^), and 2D-band (2400–1700 cm^−1^). While the G-band indicates the graphitic nature of the carbon, the D-band signifies any structural disorder or defect. The position and relative intensities of the bands conveys more information about the graphene structure (Figure 12). For example, the increase in intensity of the D-band relative to the G-band indicates defects in the structure and low-quality graphene. Moreover, from the intensity of the 2D-band relative to the G-band, the number of graphene layers can be determined. Higher 2D-band implies successful graphene exfoliation and few-layer thickness of graphene. In addition, the shape of the 2D-band can assist in identifying the number of layers in a graphene structure [65,66].

#### 2.4.4. UV-Vis Absorbance Spectroscopy

**Figure 13:**
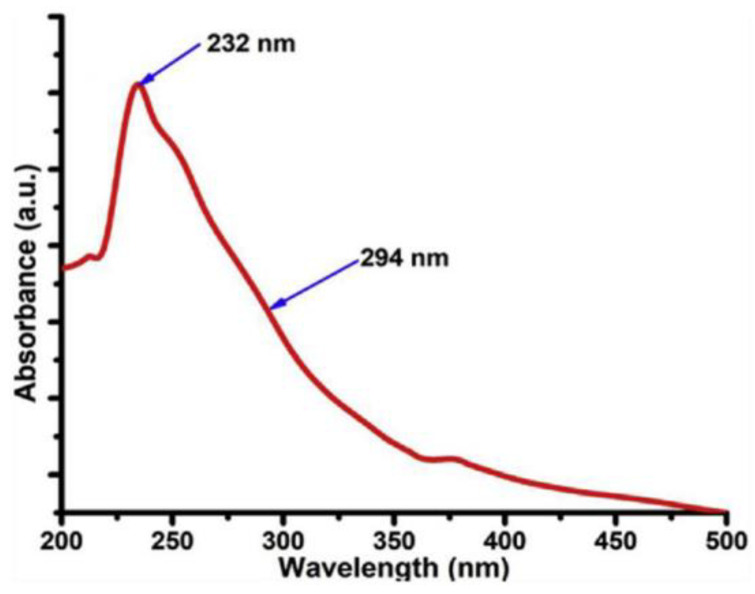
UV-Vis absorbance spectrum of graphene-oxide nanostructures. Reprinted with permission from Ref. [63]. 2019, Elsevier.

UV-Vis spectroscopy is an analytical technique that measures the absorption or transmission of discrete wavelength in the UV and visible region. Such measurements convey information regarding the composition of the sample and its concentration. Since graphene is transparent, it does not show any absorption peaks. However, other carbon materials usually exhibit two noticeable bands (Figure 13). The first band occurs in the range 260–270 nm which is assigned to the π–π* transition of C=C bond. The second band appears in the range 270–350 nm and corresponds to the n–π* transition of the C=O bond [63,67].

#### 2.4.5. Photoluminescence Emission (PL)

**Figure 14:**
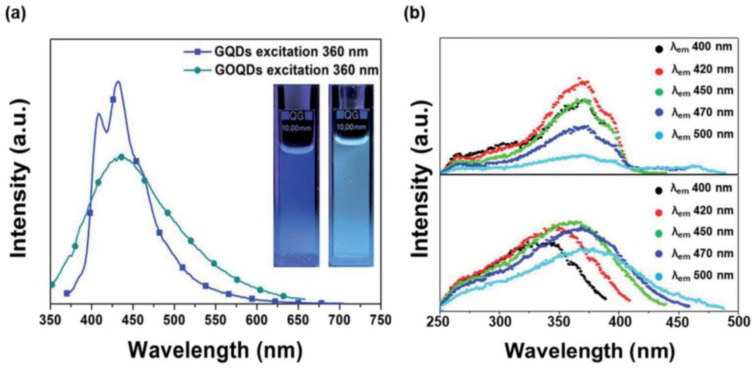
(**a**) PL spectra of GQDs and GOQDs and (**b**) their excitation dependency [68].

PL emission spectroscopy is an important technique to investigate the optical and photochemical properties of a sample. The emitted photon energies are directly related to the energy difference between orbitals and bands. Therefore, such information can assist in determining the band gap of a material. Furthermore, this method can be utilized to study the quantum confinement effect in nanostructures. One of the main characteristics of carbon nanostructures is their PL emission tunability in the 400–550 nm range. Typically, GQDs displays a distinct blue emission, whereas GOQDs exhibit a mixed emission of blue and green (Figure 14a). Furthermore, GQDs show an excitation-independent PL property for excitation wavelength 300–400 nm, while GOQDs demonstrate excitation-dependent PL properties (Figure 14b). In addition, surface functionality of the quantum dots can improve their PL emission fluorescence lifetime and intensity [68].

#### 2.4.6. Fourier Transform Infrared Spectroscopy (FT-IR)

**Figure 15:**
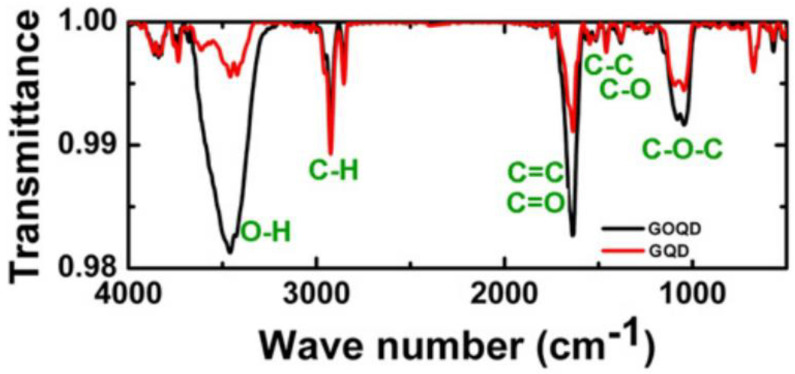
FT-IR spectrum of GQDs (red) and GOQDs (black). Reprinted with permission from Ref. [69]. 2014, IOP Publishing.

FT-IR spectra are utilized to study the different chemical bonds of materials. When analyzing the FT-IR spectra of carbon nanostructures in the 500–4000 cm^−1^ range, distinct troughs are observed. The trough positioned at 3420 cm^−1^ is related to the O–H stretching vibrational mode, 2930 cm^−1^ is ascribed to the C–H stretching, 1633 cm^−1^ is attributed to aromatic bending of C=C bonds and C=O stretches, and 1068 cm^−1^ is a characteristic of C–O–C bonds (Figure 15) [69].

## 3. Graphene Nanostructures Prepared by PLAL

**Figure 16:**
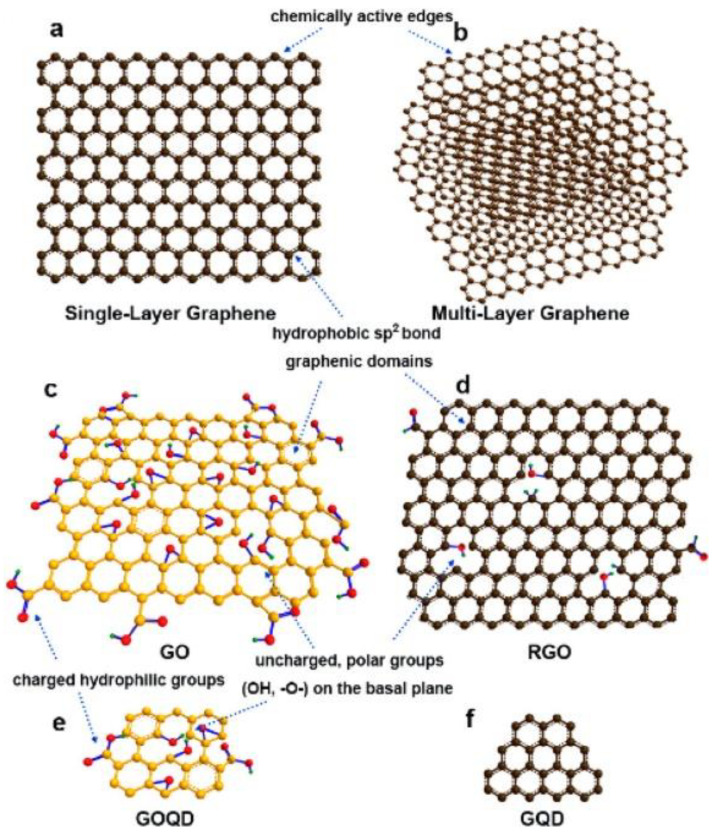
Graphene nanostructures: (**a**) single-layer graphene, (**b**) multi-layer graphene, (**c**) graphene-oxide, (**d**) reduced graphene-oxide, (**e**) graphene-oxide quantum dots, and (**f**) graphene quantum dots. Reprinted with permission from Ref. [71]. 2017, American Chemical Society.

PLAL has been used to fabricate different carbon nanostructures such as graphene nanosheets (GNS), graphene-oxide nanosheets (GONS), graphene quantum dots (GQDs), graphene-oxide quantum dots (GOQDs), graphene nanotubes (GNTs), nanodiamonds, polyynes, and carbon-encapsulating metal/metal-carbide/metal-oxide nanoparticles (C/M-CNPs) [30,70]. In this review, we will focus on GNS, GONS, GQDs, and GOQDs (Figure 16).

While graphene is a zero-band gap semiconductor, and thus does not have photoactive or photoluminescence properties, graphene oxide (GO) might have a tunable band gap depending on its oxidation level and therefore demonstrate broad photoluminescent characteristics. This feature has enabled GO to have many applications [72,73]. GO has also been used to obtain graphene nanosheets through reduction processes [74]. PLAL has been utilized to synthesize graphene and GO nanostructures.

The product obtained via a PLAL process is highly dependent on the experimental parameters used [75]. Some of these parameters are directly linked to the laser source used, such as the laser beam wavelength, its fluence, pulse repetition rate, and pulse duration. Other parameters are related to the liquid medium used, its temperature, and if there are any processes influencing the medium such as sonication and stirring. A final parameter is the laser irradiation time, which is equivalent to the number of pulses the target received during the conduct of the experiment. In the sections below, we discuss research done to fabricate graphene and GO nanostructures from the point of view of experimental parameter control.

### 3.1. Graphene and Graphene-Oxide Nanosheets

There are numerous experimental parameters that influence the formation of graphene and graphene-oxide nanosheets during a PLAL process. Below, these parameters are highlighted and their influence is discussed. A summary of the research conducted to investigate the effect of these parameters is listed in Table 2.

#### 3.1.1. Liquid Medium

For the production of graphene nanosheets through PLAL, it was reported that the properties of the liquid medium are important factors [83]. For example, the size of the molecules in the liquid medium was found to be significant, as they penetrate between the graphite target layers and cause the ablation of large graphene sheets [83]. We first concentrate on the production of graphene nanosheets when the graphite target is placed in de-ionized or distilled water as the liquid medium. The utilization of water increases the quality of graphene films by reducing the probability of impurities or defects [66].

At room temperature, graphene has been formed by irradiating a graphite target with a 532 nm Nd:YAG laser when the target was submerged in distilled water [76]. The repetition rate of the laser pulses was 10 Hz with a pulse width of 6 ns. When investigating the effect of laser fluence on the produced nanostructures, it was found that lowering the fluence to 0.4 J/cm^2^ resulted in the production of more graphene and less of other carbon nanostructures. Moreover, the study did not report the observation of graphene oxide; however, there was an indication of the presence of a small amount of oxygen trapped between graphene layers. The graphene layers were of multilayer thickness and were not stacked in an orderly fashion, as indicated by the broad peak of the XRD analysis [76].

To draw a comparison between nitrogen and water as the liquid environment for PLAL, further investigation was done [65]. The laser fluence was kept at 0.5 J/cm^2^ to favor the formation of graphene nanosheets while using different liquid environments. Different liquid environments result in different pressures of the plasma plume on the surface of the target. This pressure is found to affect the structure, size, and morphology of the formulated nanostructures and their aggregation. A decreased plasma plume pressure favors the formation of graphene nanosheets when graphite targets are used in PLAL experiments. To determine the quality of the produced graphene nanosheets when obtained from different liquid environments, Raman spectroscopy was performed. It was found that I_D_/I_G_ = 0.25 when nitrogen was used as a liquid medium, while I_D_/I_G_ = 0.94 when distilled water was used. Since the D band is related with structural defects, a lower I_D_ indicates a better quality. Hence, it was concluded that using nitrogen as a liquid environment produced better-quality graphene. The number of graphene layers could also be concluded from Raman spectroscopy by calculating the ratio of the 2D band intensity to that of the G band. The use of liquid nitrogen resulted in the formation of fewer layers of graphene when compared to the use of distilled water. However, when XRD analysis was performed, the samples obtained with distilled water did not show a graphite peak at 26.58°, which is an indication of the presence of graphene. Samples obtained with liquid nitrogen also did not show any peak at 26.58°; however, they showed a sharp peak at 32.1°. This could indicate the presence of a great proportion of other carbon nanostructures in the colloidal solution [65].

Cryogenic liquids have also been employed for graphene nanosheet production via PLAL. Liquid nitrogen was used to generate graphene nanosheets using PLAL [83]. A 1064 nm Nd:YAG laser was used with 10 ns pulse duration, 5 Hz repetition rate, and 6 J/cm^2^ fluence to irradiate a graphite target immersed in liquid nitrogen. The target and liquid nitrogen were contained in a cell with a BK7 window in front of the laser beam. The laser beam was focused using a concave lens to a 1 mm^2^ spot on the target. After irradiation, the liquid nitrogen was evaporated at room temperature and de-ionized water was added to provide a suspension for the graphene nanosheets. It was suggested in this work that the cryogenic liquid forms a 2D-fluid monolayer which can penetrate the spacing of graphite. When laser exposure takes place, the liquid molecules expand to a gaseous phase where it supports the dissociation of the weak bonds of graphite layers. Hence, graphene nanosheets are formed. Single and bi-layer graphene sheets were found in the suspension along with graphite films and nanodiamonds. The Raman analysis of the D and G bands concluded that the quality of the graphene nanosheets was acceptable (I_D_/I_G_ ≈ 0.5) [83].

Porous graphene (PG) has been synthesized using a highly oriented pyrolytic graphite (HOPG) target submerged in water and irradiated by a 35 fs laser with wavelength of 800 nm and 1 kHz repetition rate. The fluence was set in the range from 20–30 J/cm^2^. The PG was six layers thick and had porous dimensions of ~20 nm. When liquid nitrogen was used instead of water, while keeping all other experimental parameters the same, no PG was observed. It was concluded that O_2_ and H_2_O_2_ are key reagents for the formation of PG [82].

**Figure 17:**
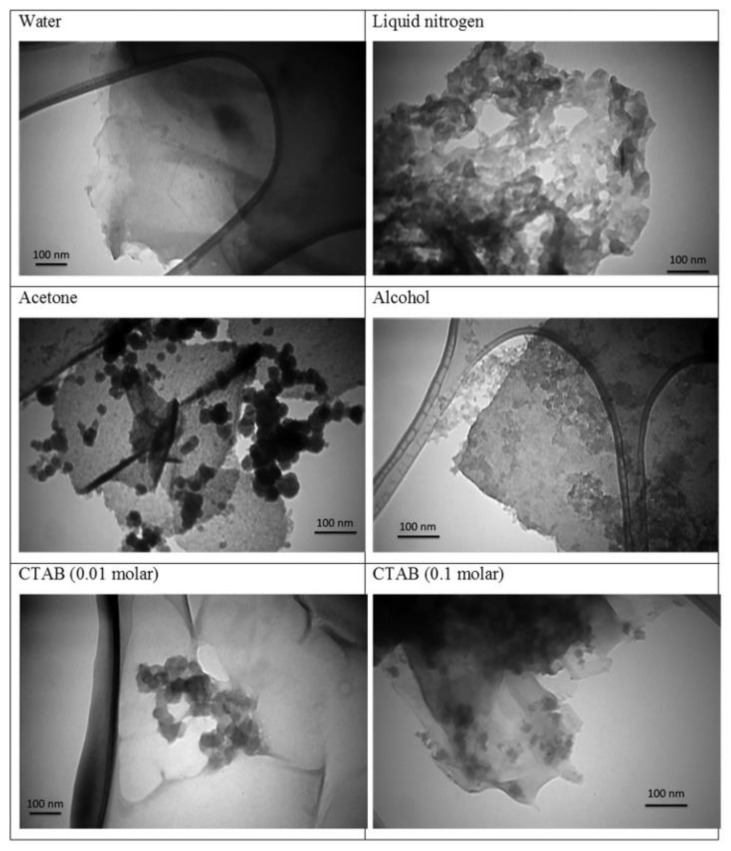
The effect of liquid medium on the formation of GNS. Reprinted with permission from Ref. [85]. 2019, Laser Institute of America.

Graphene nanosheets were also produced using PLAL with liquid media other than water. In order to investigate the effectiveness of water against other liquids as an ablation medium, liquid nitrogen, acetone, alcohol, and (0.01 M and 0.1 M) of cetyl trimethylammonium bromide (CTAB) were used [84,85]. By immersing a graphite plate in the different liquid media and keeping all experimental parameters fixed, the effect of different liquid mediums was investigated. A 7 ns laser pulse with 5 Hz repetition rate of the fundamental 1064 nm wavelength of an Nd:YAG laser was focused on the graphite plate with a fluence of 1.5 J/cm^2^, and 5000 laser pulses were shot on the target. In each sample, graphene nanosheets and carbon nanoparticles were found but with different percentages. Where CTAB contained the least amount of graphene sheets, this amount increased in alcohol, acetone, and liquid nitrogen, and was the highest in water (Figure 17). However, in liquid nitrogen, the graphene nanosheets had few layers when compared with those obtained from water. Moreover, in acetone, the size of the nanosheets was the greatest. This was concluded from the analysis of SPR peak, Raman spectroscopy, and TEM images. The liquid medium used in the ablation process affected the pressure on the generated plasma plume. The pressure, in turn, affected the structure, size, and morphology of the produced nanostructures. Another point that needs to be considered regarding the liquid used is the size of the liquid molecules. For example, in graphite, the spacing between the planes is on the order of the size of the water molecules, making water more effective in penetrating the graphite planes and separating the graphene sheets [84,85].

Although (0.01 Molar) CTAB, when used as an ablation liquid medium, showed the lowest concentration of graphene nanosheets [84], the effect of its concentration was further investigated [62]. An Nd:YAG laser of 1064 nm with a pulse width of 7 ns and 1.5 J/cm^2^ fluence was focused on a graphite target submerged in CTAB of different concentrations. The main advantage of using CTAB as an ablation medium is that it decreases the aggregation of the produced nanostructures and hence increases their stability. However, it was found that different concentrations of CTAB can affect the properties of the produced graphene. By increasing the concentration of CTAB from 0.02 to 0.1 M, a critical concentration seemed to exist where the quality of the produced graphene was the highest. This critical concentration was about 0.06 M. Above the critical concentration, the liquid medium became denser, causing the pressure on the plasma plume to increase. This increased pressure resulted in the breakage of the produced graphene and increased its aggregation. Below the critical CTAB concentration, the produced graphene nanosheets were less aggregated and the thickness of the graphene nanosheets decreased. However, decreasing the CTAB concentration even more caused the nanosheet to be thicker. A similar investigation was performed using a 0.04 M concentration of CTAB with a 532 nm laser wavelength and a reduced laser fluence of 0.5 J/cm^2^. It was found that graphene nanosheets obtained had a high level of defects and were smaller in size; the ratio *I_D_*/*I_G_* reached 1.93, indicating poor quality.

Pulsed laser exfoliation of HOPG in liquid has also been used with liquids other than water. For example, *N*-methyl-pyrrolidone (NMP) and sodium dodecylbenzene sulfonate/water were used as ablation liquid media [77]. It was found that these media help in graphene dispersion and prevent oxidation. An Nd:YAG laser of wavelength 532 nm, with 5 Hz repetition rate and 7 ns pulse duration, was focused on an HOPG target that was submerged in the liquid medium and irradiated for 2 h. The laser fluence was 1.0 J/cm^2^. The suspension was left for 24 h and then centrifuged for 20 min at 500 rpm. The upper uniform graphene suspension was characterized and showed the existence of graphene sheets of ~1 nm in thickness (three-layer graphene) and tens of micrometers in size.

Furthermore, a three-liquid medium was tested to investigate its effect on fabricated graphene nanosheets. Flexible graphite target was submerged in de-ionized (DI) water, acetone, and dimethylformamide (DMF) and was irradiated under a 532 nm wavelength, 50 mJ/pulse energy, and 5 Hz repetition rate for a irradiation time of 5 min [89]. Results showed that acetone produced high-quality graphene nanosheets when compared with other media, with no nanoparticles present on the nanosheets. This was attributed to the high molecular dipole moment of acetone, which increased the repulsive force between graphene layers.

In PLAL, when the ablation medium has plenty of oxygen, there will be a higher probability that the carbon atoms will be oxidized during the production of graphene [78]. Upon applying a 532 nm green laser at 7 ns pulse length, 5 Hz repetition rate, and 0.7 J/cm^2^ fluence to a graphite plate immersed in different liquids for 5000 laser pulses, graphene, graphene oxide, and carbon nanoparticles were produced. By keeping the experimental conditions fixed and only changing the ablation medium, it was possible to investigate the best conditions for obtaining graphene-oxide nanosheets. By comparing de-ionized water, liquid nitrogen, and 0.01 M CTAB, it was found that de-ionized water produced better-quality GO nanosheets that are large and least in defects compared to the other liquids [78].

**Figure 18:**
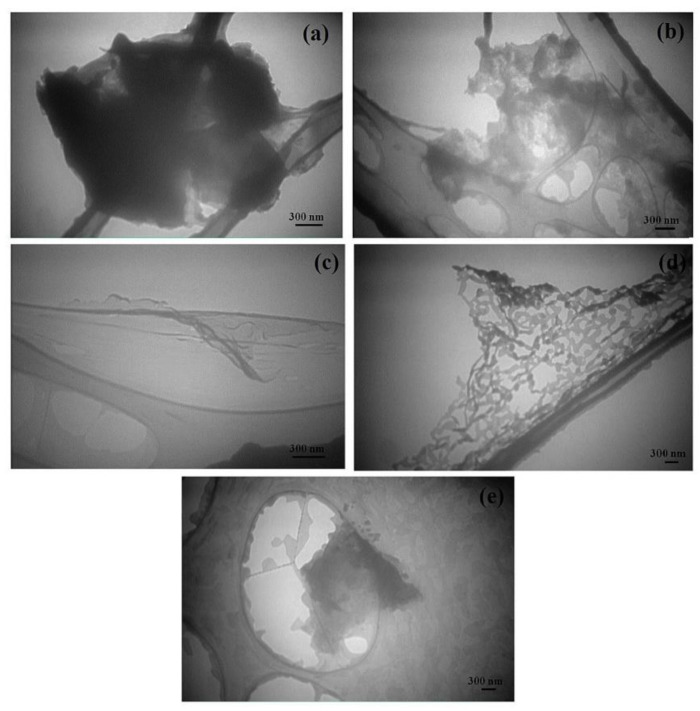
TEM images of GO nanosheets in different concentrations of CTAB: (**a**) 0.02 M, (**b**) 0.04 M, (**c**) 0.06 M, (**d**) 0.08 M, and (**e**) 0.1 M. Reprinted with permission from Ref. [79]. 2018, Elsevier.

To further investigate the optimized conditions for producing GO nanosheets, the effect of different concentrations of CTAB as a liquid medium was investigated [79]. The emphasis on CTAB is due to the possibility of improving the problem of graphene-oxide nanosheet aggregation. It was concluded that to improve the quality of the GO nanosheets, there should be a balance between the negative charge of the oxygen functional groups and the positive charge of CTAB molecules. This balance was reached when the concentration of CTAB was 0.04 M [79] (Figure 18). When comparing 0.04 M CTAB as a liquid medium with liquid nitrogen [80], it was found that liquid nitrogen produced higher-quality multilayer graphene, while 0.04 M CTAB randomly stacked multilayer GO nanosheets.

#### 3.1.2. Liquid Temperature

**Figure 19:**
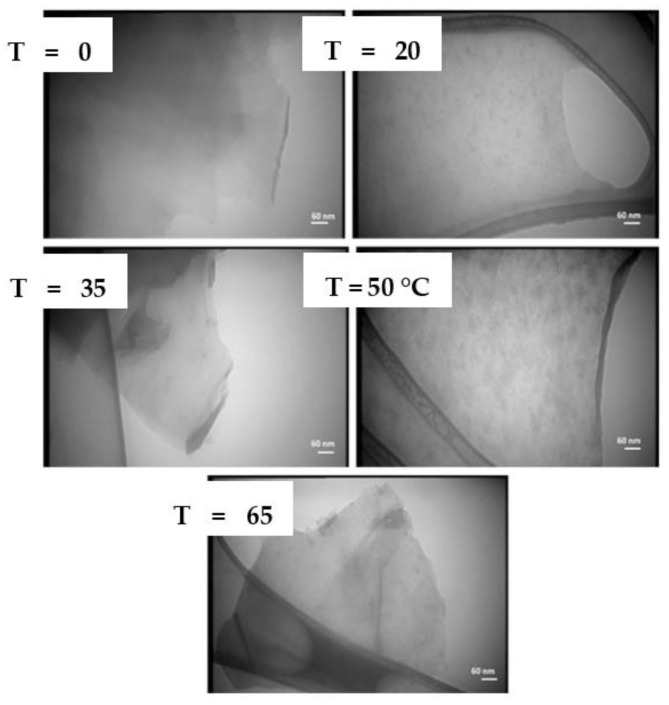
The effect of liquid temperature on the formation of GNS [57].

The effect of liquid temperature on the quality of the production of graphene through PLAL was investigated [57]. A 1064 nm pulsed Nd:YAG laser with 7 ns pulse width and 5 Hz repetition rate was used to irradiate a graphite target submerged in distilled water. The 6 mm diameter laser beam was focused on the target using a convex lens of 80 mm focal length. The fluence of the laser pulse was 0.6 J/cm^2^ and 5000 laser pulses were aimed at the target. Five samples were prepared, where the distilled water of each sample was kept at a different temperature. These temperatures were 0, 20, 35, 50, and 65 °C. All samples showed the formation of graphene and carbon nanostructures with different proportions. The low-temperature samples contained more graphene nanosheets while the high-temperature samples had more carbon spherical nanoparticles. Furthermore, the XRD analysis showed that increasing the temperature of the liquid medium resulted in better crystallinity of the produced nanoparticles and a deviation from the graphene monolayer structure. The absorption peaks of the samples in the UV–Vis and FT-IR analysis were not affected by the change in temperature. However, the intensity of the absorption in both analyses increased as the temperature increased. This was attributed to the increase of the number of nanostructures with increased temperature. This investigation concluded that to obtain large and smooth nanosheets of graphene with few layers, the distilled water must be kept at 0 °C. Liquid temperature in the range of 35 °C gave thicker graphene sheets. When the temperature was increased even more, the graphene sheets became more chopped and more spherical carbon nanoparticles were formed [57] (Figure 19).

#### 3.1.3. Laser Wavelength

Laser wavelength in the PLAL process may have an effect on the produced nanostructures. To investigate the effect of laser wavelength on the production of graphene nanosheets, two wavelengths, 532 nm and 1064 nm, were compared while using liquid nitrogen as the ablation medium [90]. The wavelength of the laser beam has a great effect on the nature of the ablation process in PLAL, which in turn influences the produced nanostructures. For example, shorter wavelengths of high photon energy involve fragmentation of the target material and hence follow a top-down approach. Meanwhile, longer wavelengths of low photon energy are more relevant to the inverse Bremsstrahlung (IB) process, followed by a bottom-up method. Moreover, the ablation rate was found to increase with decreasing wavelength, and this can be confirmed by the increased absorption when employing UV–Vis spectroscopy. It was observed that using 1064 nm as the ablation wavelength of a graphite target resulted in the production of graphene nanosheets. Decreasing the wavelength to 532 nm led to more ablation, which included graphene nanosheets and spherical carbon nanoparticles [90].

**Figure 20:**
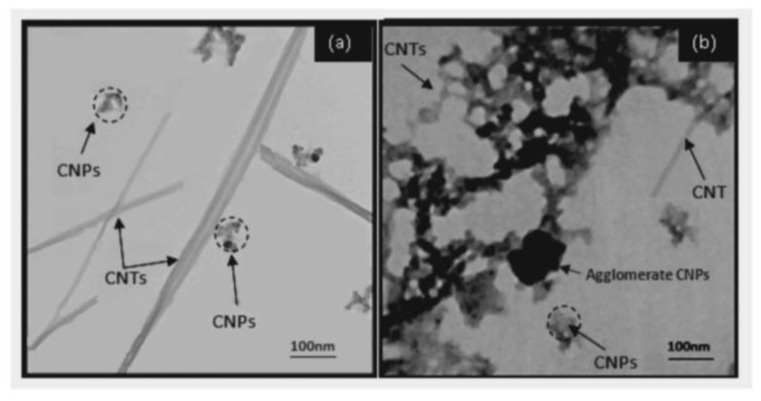
The effect of laser wavelength on the formation of GNS: (**a**) 532 nm and (**b**) 1064 nm. Reprinted with permission from Ref. [12]. 2020, Elsevier.

To investigate the effect of wavelength on the produced carbon nanostructures, a graphite pallet was placed in the bottom of a glass beaker filled with double distilled water and irradiated with 532 nm and 1064 nm beams [12]. The laser had a pulse duration of 9 ns, repetition rate of 2 Hz, and fluence of 15.4 J/cm^2^. The target was irradiated for 30 min while rotating the sample throughout. By examining the colloidal solution, it was found that it contained carbon nanotubes (CNT) and carbon nanoparticles (CNPs). The wavelength of the laser beam affected the size of the produced CNTs: they had an average diameter of 26 nm when the 532 nm laser was used and 35 nm when the laser with a wavelength of 1064 nm was used. Moreover, the concentration of the fabricated CNT was higher for 1064 nm compared to 532 nm. This was attributed to the absorption depth, which was small for 532 nm and high for 1064 nm, hence, smaller-size nanostructures were fabricated with the 532 nm beam (Figure 20). Furthermore, the bandgap of the produced CNT was related to the laser wavelength employed: 3.33 eV when a 1064 nm laser was used and 3.5 eV with the 532 nm laser. The CNPs were found to be adhered randomly to the surface of the CNTs. The quality of the CNTs that were produced with the 1064 nm wavelength were higher compared to those produced with the 532 nm wavelength, as the Raman analysis indicated [12].

Two more important points that were investigated are the quality of the produced graphene nanosheets and the role of the wavelength on the quality and thickness of the nanosheets [90]. The quality of the graphene nanosheets is measured through the ratio of the intensities of the D and G bands (I_D_/I_G_), which indicates the level of disorders. As for the thickness of the graphene nanosheets, it is usually found through the ratio of the intensities of the 2D and G bands (I_2D_/I_G_). It was concluded that the best quality was achieved when using 532 nm laser beam; however, the sheets were of multilayer thickness. This was also confirmed by other work by the same group [56]. Bilayer graphene nanosheets were achieved when increasing the wavelength to 1064 nm, but this was at the expense of the quality of the produced nanosheets.

#### 3.1.4. Laser Fluence

**Figure 21:**
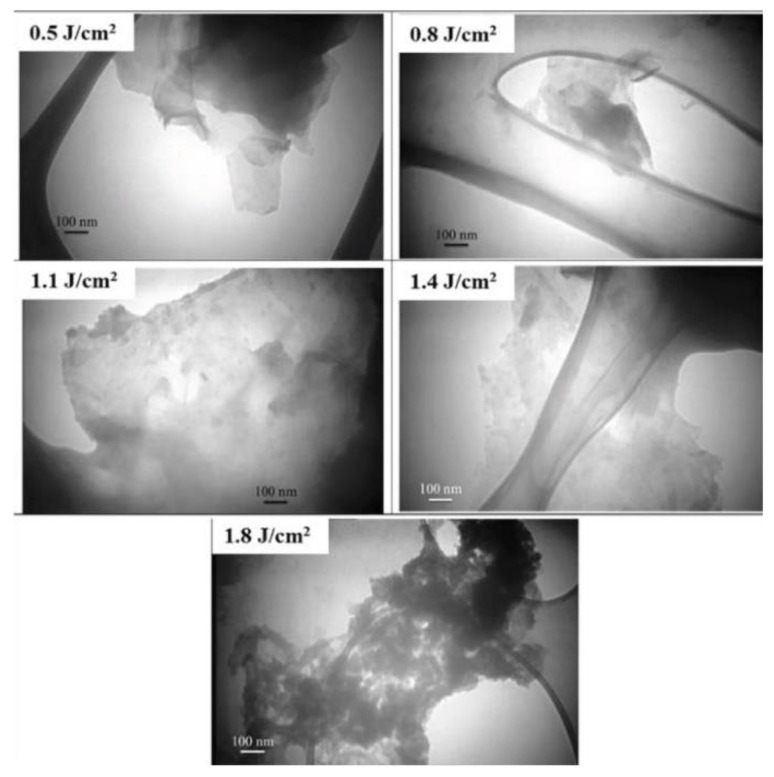
The effect of laser fluence on the formation of GNS [86].

The effect of laser fluence on the production of graphene nanosheets via PLAL when graphite target is immersed in liquid nitrogen was studied [86] (Figure 21). It was found that the formation of nanosheets and nanoparticles are highly influenced by the laser fluence. Lower laser fluence resulted in the dominance of nanosheets, whereas higher laser fluence assisted the formation of more nanoparticles. Hence, the competition between nanosheets and nanoparticles formation is controlled by laser fluence. It was concluded that a fluence of 1.1 J/cm^2^ is approximately the borderline between nanosheet and nanoparticle formation when a 1064 nm laser wavelength is used [86].

The quality and thickness of graphene nanosheets under different fluences were investigated [90]. By comparing the fluence of 0.5 J/cm^2^ and 0.8 J/cm^2^ for a laser wavelength of 532 nm, it was found that increase in laser fluence resulted in the production of more carbon nanostructures in comparison to graphene nanosheets. Moreover, increase in fluence from 0.5 J/cm^2^ to 0.8 J/cm^2^ when a 1064 nm laser was employed caused the graphene nanosheets to be torn and fragmented [90].

Another investigation was performed using a 532 nm laser beam focused on a graphite target with laser fluences of 0.5, 2.2, and 3.6 J/cm^2^, 15 min of irradiation time, and ultrasonic wave assistance [55]. It was found that the number of layers in the graphene nanosheets increased with increasing laser fluence. Moreover, 0.5 J/cm^2^ gave the thinnest nanosheets, ranging from a single layer up to four layers.

In order to determine the threshold fluence for the formation of PG while using a femtosecond laser to irradiate an HOPG target in water, it was found that a laser fluence below 12 J/cm^2^ cannot result in the formation of PG. A fluence in the range of 20–30 J/cm^2^ is required for the formation of PG layers [82].

A pulsed Nd:YAG laser of 1064 nm wavelength and pulse duration of 7 ns was employed in a PLAL process that involved post-irradiation [87]. Two laser energies, 80 mJ and 160 mJ, were used to irradiate a graphite target immersed in water. After 100 pulses, the target was removed and the colloidal solution was re-irradiated with the same energy for an additional 100 pulses. It was found that straight, isolated, lone multiwall carbon nanotubes (MWCNTs) were formed with hollow core. The diameter of the MWCNTs was larger (66–75 nm) under a pulse energy of 160 mJ compared to the diameter under 80 mJ (25–30 nm). After re-irradiation, the MWCNTs were unzipped to form graphene nanosheets with slight folds and wrinkles under a pulse energy of 80 mJ. When irradiating with 160 mJ, carbon nanoparticles were formed along with PG nanosheets with pore sizes ranging from 7–16 nm. It was concluded that increasing the laser energy resulted in the re-arrangement of carbon atoms to build other forms of carbon nanostructures.

Most PLAL processes incorporate lasers of wavelength 1064 nm and 532 nm. Rarely, wavelengths of 355 nm and 266 nm are also used. To investigate the effect of wavelength and fluence on the production of graphene oxide, these parameters were changed in four experiments (6.61 J/cm^2^ @1064, 3.33 J/cm^2^ @1064, 1.33 J/cm^2^ @532, and 0.38 J/cm^2^ @266 nm) [91]. The pulse duration and repetition rate were kept constant in all experiments at the values of 18 ns and 10 Hz, respectively. The laser beam was focused on a graphite target immersed in doubly distilled water, where the liquid layer was 6 mm above the target. It was concluded that the laser fluence had a great effect, where lower fluence resulted in the production of graphene-like phases (FLG and folded FLG). On the other hand, higher fluence opted the production of rGO and GO particles. However, it was observed that the production of rGO decreased with increasing photon energy. Hence, the influence of photon energy cannot be neglected and requires further investigation.

#### 3.1.5. Irradiation Time/Pulse Duration/Repetition Rate

Using a 532 nm wavelength, a graphite plate submerged in acetone was exfoliated to produce graphene nanosheets [58]. Using a laser fluence of 0.5 J/mm^2^ and a pulse width of 7 ns, the laser beam irradiated the graphite target for different time intervals of 10, 20, 30, 40, and 60 s. It was found that the irradiation time has an effect on the produced carbon nanostructures. At 10 s of irradiation, bi-layered graphene was obtained. When the irradiation time was increased to greater than 30 s, the colloidal solution was mainly amorphous carbon and graphite.

**Figure 22:**
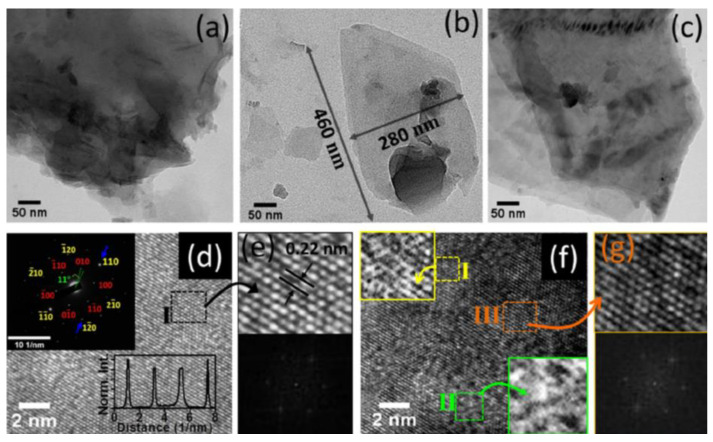
The effect of irradiation time on the formation of GNS: (**a**) t = 20 min, (**b**) 30 min, (**c**) 50 min, (**d**) HRTEM for t = 30 min sample and its FFT (**e**), (**f**) HRTEM for t = 50 min sample and its FFT (**g**). Reprinted with permission from Ref. [88]. 2020, Elsevier.

Furthermore, to investigate the effect of irradiation time on the formation of graphene nanosheets via PLAL method, dry-cell graphite electrodes (DGE) were used as a graphite source [88]. After transforming the bulk graphite into powder, the powder was dispersed in an aqueous solution and irradiated with a 1064 nm laser beam with a repetition rate of 10 Hz and 10 ns pulse duration. The laser fluence was kept constant at 60 J/cm^2^, while the irradiation time varied from 20 to 50 min. It was found that 20 min of irradiation was not sufficient for graphene exfoliation, while 30 min was the best irradiation interval to obtain bi-layer graphene, which is highly crystalline. Extending the irradiation time to 50 min resulted in the formation of multi-layer graphene (Figure 22). This work illustrated the ability to use waste material in a PLAL method to synthesize bi-layer graphene, which could reduce the cost of its production [88].

#### 3.1.6. Target Material

**Figure 23:**
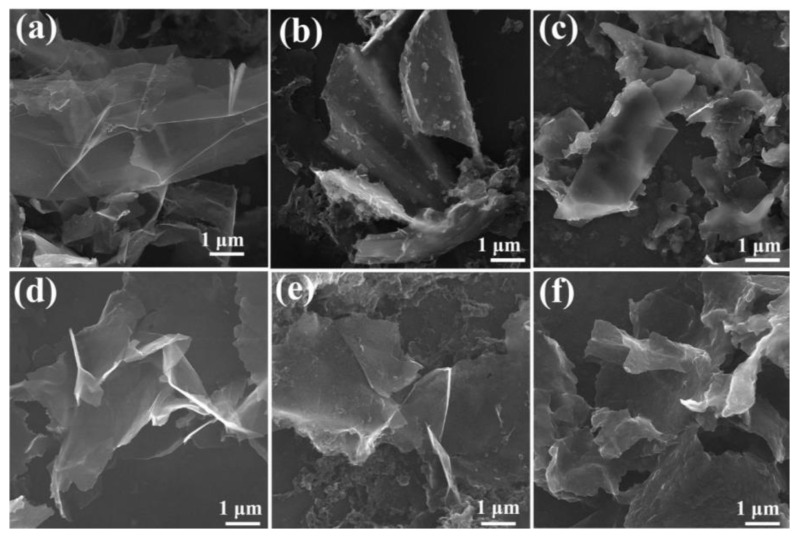
The effect of target material on the formation of GNS: (**a**) FG1 in acetone, (**b**) FG1 in water, (**c**) FG1 in DMF, (**d**) FG2 in acetone, (**e**) FG2 in water, (**f**) FG2 in DMF. Reprinted with permission from Ref. [89]. 2019, Elsevier.

Different graphite targets have shown to have an effect in PLAL [89]. When comparing the graphene nanosheets produced from flexible graphite targets, FG1 (96% carbon, 1.6% SiO_2_, 0.1% K_2_O, and 0.7% Al_2_O_3_), FG2 (84% carbon, 0.2% Cr, 0.8% CaO, and 9% SiO_2_), and a nuclear graphite (NG), it was found that NG did not produce graphene nanosheets. Moreover, FG1 resulted in fewer defects in the produced graphene nanosheets. The quality of the prepared graphene nanosheets as determined from I_D_/I_G_ showed that FG1 produced a better quality with a ratio of 0.3 compared to 0.4 when FG2 was used, with the number of layers being less than ten layers [89] (Figure 23).

#### 3.1.7. Extending PLAL Technique

**Figure 24:**
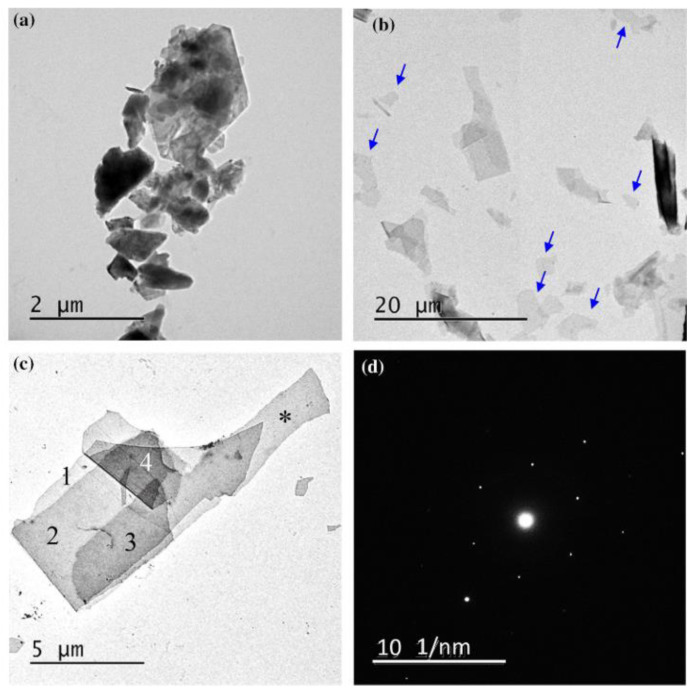
The effect of sonication integrated with PLAL. Carbon nanostructures (**a**) without sonication, (**b**) with sonication, (**c**) zoom of b, (**d**) electron diffraction pattern of a single-layer graphene indicated by an asterisk in (**c**) [55].

PLAL has proved to be a good method for producing graphene nanosheets. Work has been done to investigate the outcome of combining PLAL with other techniques. The effect of applied ultrasonic waves during a PLAL process has been studied [55]. A 532 nm laser beam was focused on the surface of a graphite target placed in a container and covered with distilled water. The container was then placed in an ultrasonic bath operating at a frequency of 40 kHz and power of 70 W. The fluence of the laser beam took different values (0.5, 2.2, and 3.6 J/cm^2^), and samples were irradiated for 15 min. When comparing samples that were placed in an ultrasonic bath with those that were not, it was found that the presence of ultrasonic waves had a significant effect. Incorporating ultrasonic waves during the PLAL process assisted in the exfoliation of graphene nanosheets. These sheets were large in size and regularly shaped. Moreover, these nanosheets were single-layer and up to four layers, rarely containing few layers (Figure 24). The quality of the graphene nanosheets was improved when ultrasonic waves were applied as illustrated by the intensities of the D and G bands in the Raman spectra [55].

**Figure 25:**
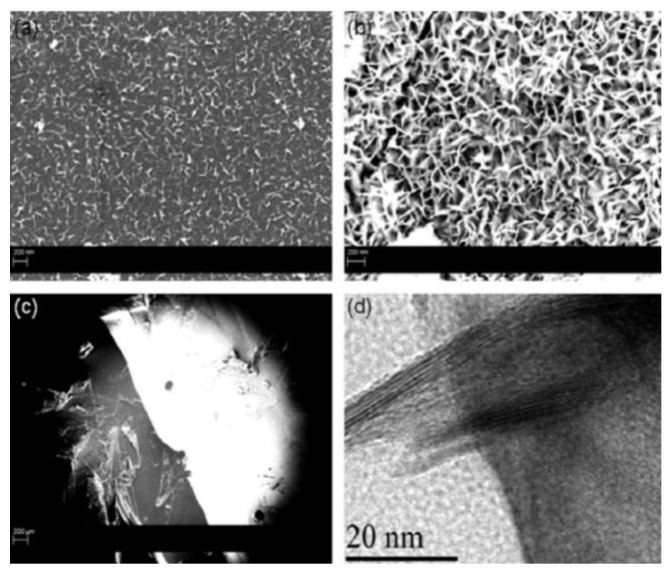
Electric-field-assisted laser ablation in liquids. SEM of CNW deposited after (**a**) 20 min and (**b**) 2 h ablation. (**c**) The dipping interface and (**d**) an HRTEM image of the walls. Reprinted with permission from Ref. [54]. 2012, Elsevier.

Taking the formation of graphene a step further, some work has been done where laser ablation of graphite in water was assisted with an electric field to form few-layer graphene nanowalls [54]. The electric-field-assisted laser ablation in liquids (ELAL) technique was used to provide a competitive method that is simpler and more economical than other methods used to fabricate graphene nanowalls, such as CVD. Two HOPG electrodes were immersed in a flask of water in which a polycrystalline graphite target was fixed at its bottom. A Nd:YAG laser beam of 532 nm wavelength, 5 ns pulse duration, and 10 Hz repetition rate was focused on the graphite target with fluence of 5 J/cm^2^. The two electrodes were charged to 30 V DC and irradiation time varied from a few minutes up to 2 h. It was found that the positive electrode showed a deposition of flower-like graphene nanowalls with thickness of 2–6 layers [54] (Figure 25).

**Figure 26:**
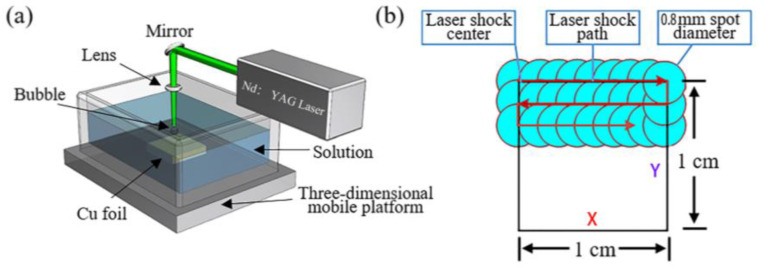
(**a**) Experimental setup of laser deposition by PLAL. (**b**) Laser scan path [32].

**Figure 27:**
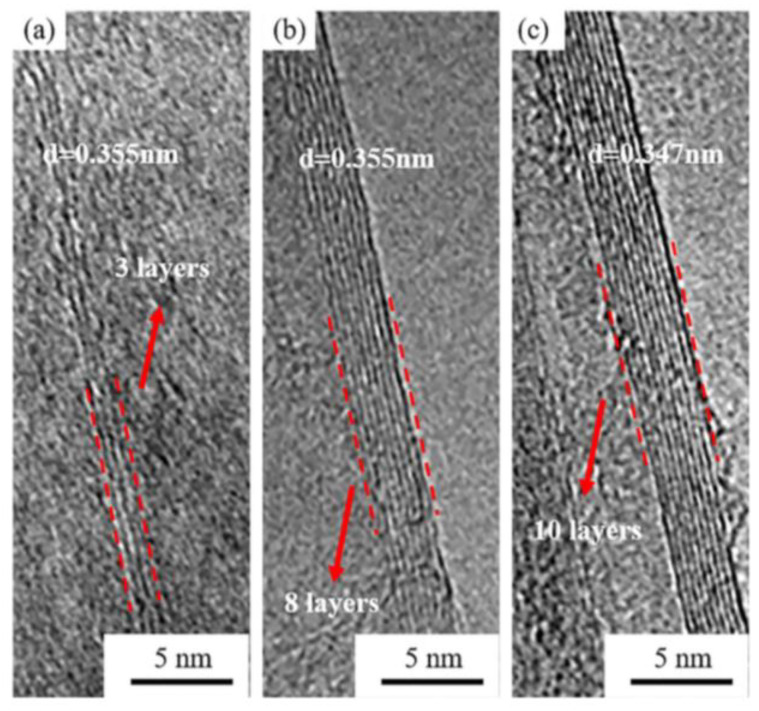
HRTEM images of FLG for laser energy: (**a**) 0.1 J, (**b**) 0.2 J, and (**c**) 0.3 J [32].

When graphene is formed in a colloidal solution by PLAL, it is usually followed up by steps to separate and purify it. These steps result in lowering the yield of the fabricated graphene layers. To overcome this limitation, work has been done to directly deposit few-layer graphene (FLG) on a foil using the pulsed ablation in liquid technique. An attempt to directly deposit FLG on a Cu foil using PLAL to study the effect of laser energy on the structure and number of layers of the FLG was performed [32]. Graphite flakes were dispersed in de-ionized water so that the suspension would be of specific concentration. A polished Cu foil was placed 3–4 mm at the bottom of the container of the solution and the pulsed laser beam was used to irradiate the target from a vertical orientation using an Nd:YAG laser of wavelength 1064 nm. The repetition rate of the pulses was 10 Hz with a pulse width of 10 ns. The container was placed on an XYZ mobile platform in order for the laser beam to map the surface of the foil while the graphite suspension was stirred with a magnetic stirrer. A schematic figure of the experimental set up is shown in Figure 26 [32]. The laser energy was varied from 0.1 to 0.4 J in steps of 0.1 J. After the completion of the ablation process, the foil was rinsed and dried. The results of this work showed that at lower laser energy, a few pieces of FLG were formed that were as low as three graphene layers in thickness. As the laser energy increased, the lateral size of the FLG increased, reaching up to 100 μm, and the number of graphene layers increased as well, reaching 10 layers (Figure 27). At higher laser energy, the Cu foil was itself ablated. Hence, it was concluded that the number of layers of graphene can be controlled by the adjustment of the laser energy [32].

Bilayer graphene (BLG) films on a Si/SiO_2_ substrate using PLAL were accomplished [66], where a KrF laser of wavelength 248 nm was used. This wavelength was chosen in particular as its photon energy is about 5 eV, which is much higher than the photons of the Nd:YAG 1064 nm laser, which is only 1.16 eV. This higher photon energy is able to break the C–C bonds and not merely cause a photothermal effect. However, unlike other work [32], the substrate was not placed in the same cell as the graphite during the ablation. After the graphite target was ablated while being immersed in DI water, the Si/SiO_2_ was dipped in the colloidal solution using a tweezer and then taken out and dried naturally. An extensive Raman spectroscopy was employed to confirm the formation of BLG. It is also worth noting that the fluence of the laser beam had a great effect on the number of graphene layers. Increasing the laser fluence increased the number of graphene layers on the substrate. It was found that a BLG was obtained with a fluence of 2.5 J/cm^2^ [66].

### 3.2. Graphene and Graphene-Oxide Quantum Dots

As seen above, PLAL can be used to fabricated graphene nanosheets; however, it can also be used to fabricate graphene nanoparticles. It was found that by increasing the laser fluence, nanoparticles can be formed. When it comes to terminology, there seems to be a wide range of names that indicate nano carbon particles. These names include carbon nanoparticles (CNPs), carbon dots (CDs), graphene quantum dots (GQDs), and graphene-oxide quantum dots (GOQDs). The name is usually linked to the type of carbon precursor used and the structure and properties of the produced nanostructure [40].

Graphene quantum dots (GQDs) have emerged as a beneficial derivative of graphene that is more suitable for applications such as photovoltaics, transistors, and light-emitting diodes due to its tunable band gap feature as a result of the quantum confinement effect [92,93,94]. GQDs have sizes not exceeding 10 nm with thickness ranging from one to five layers of graphene. The mechanism for the formation of GQDs from MWCNTs by PLAL has been investigated theoretically and experimentally. It was found that the formation of GQDs is strongly dependent on the laser pulse energy [38]. There are many advantages of GQDs, as they are environmentally friendly, non-toxic, and show photoluminescence with broad emission regions [93]. The effect of experimental parameters on the synthesis of GQDs and GOQDs is discussed below and summarized in Table 3.

#### 3.2.1. Liquid Medium

Carbon particles with flower-like morphology have been fabricated using 1064 nm laser with 2 Hz repetition rate, 5 ms pulse duration, and 3 J/pulse of energy [107]. A graphite target submerged in ethanol was irradiated with a laser beam that was first focused with a 5 cm lens. After 5000 pulses, the graphite target was removed, and an extra 25,000 pulses irradiated the colloidal solution while being constantly stirred. The produced particles had a broad size distribution from 200–500 nm and exhibited fluorescent properties by emitting blue-green color when irradiated by UV light. When the same experiment was repeated while changing the liquid medium to DMF, the produced particles changed shape to spherical with a reduced size ranging from 80–130 nm [108].

A pure glassy carbon plate was submerged in de-ionized water and irradiated with a 532 nm Nd:YAG laser [100]. The laser pulses were 7 ns in duration and the repetition rate was 10 Hz. The fluence of the laser beam was set at 0.8 J/cm^2^ and the sample was irradiated for 5 min. The nanoparticles that were fabricated with these experimental settings had sizes ranging from 10 nm to 20 nm with a few particles having sizes larger than 20 nm. The selected area diffraction (SAD) showed that the structure of the nanoparticles was amorphous and proved to have optical limiting properties. This investigation was taken further by using a liquid other than water. When tetrahydrofuran was used with laser fluence of 1 J/cm^2^ and irradiation time of 30 min, the size of the produced carbon nanoparticles was reduced, reaching an average of 6 nm [101]. Hence, the choice of the ablating liquid medium has a great effect on the size of the resulting nanoparticles.

GQDs were synthesized using PLAL [109]. By using a 1064 nm laser with 40 mJ/pulse, 6 nm pulse duration, and 10 Hz, a HOPG target was irradiated while placed in solution consisting of 5 mL of polyethylene glycol (PEG) and 5 mL of water. After 30 min of irradiation, the solution was centrifuged to remove graphene sheets and large particles to obtain GQDs remaining in the suspension. The size of the produced GQDs ranged from 2 to 10 nm. Furthermore, the solution was also refluxed at 200 °C for 20 min and 1 hr to investigate its effect on the fluorescence emission of the GQDs; it was found that 20 min produced GQDs of strongest fluorescence intensity.

Using a femtosecond laser with pulse duration of 35 fs, 800 nm wavelength, and 1 kHz repetition rate, an HOPG target was submerged in water and irradiated for 20 min with laser fluence ranging from 20–30 J/cm^2^. Investigating the colloidal solution obtained showed the existence GQDs. The GQDs were of 2–5 nm size. To investigate the effect of liquid medium on the formation of the GQDs, water was replaced with liquid nitrogen. Although all experimental parameters were kept the same, no GQDs were observed in this case. Hence, it was shown that one of the main factors for the formation of GQDs is the presence of O_2_ and H_2_O_2_ in the liquid medium [82].

**Figure 28:**
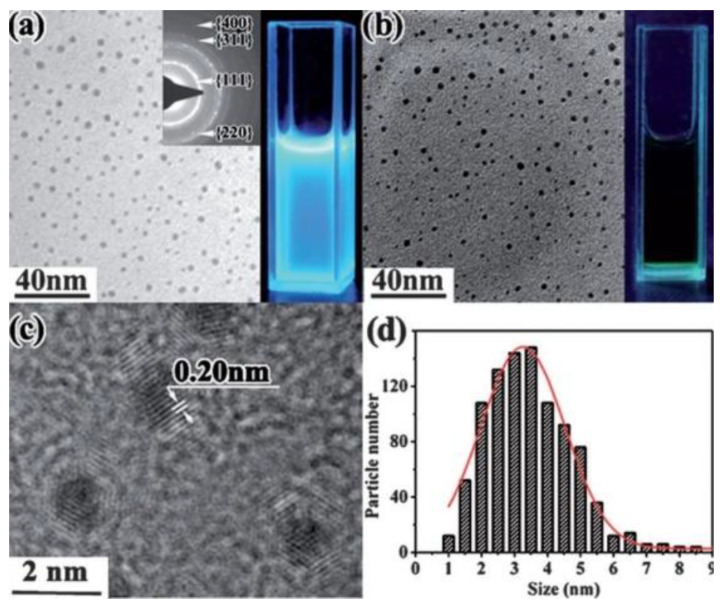
TEM images of CNPs prepared in (**a**) PEG and (**b**) water. (**c**) HRTEM of CNPs prepared in PEG and (**d**) their size distribution. Reprinted with permission from Ref. [110]. 2009, Royal Society of Chemistry.

Starting with graphite powder dispersed in water and PEG_200N_, PLAL was performed using a 1064 nm laser with power density of 6.0 × 10^6^ W/cm^2^. After 2 h irradiation, carbon nanoparticles were obtained. The size of the CNPs were in the range of 3 nm with a narrow size distribution. Moreover, the CNPs prepared in PEG_200N_ displayed photoluminescence properties, irradiating blue light when excited by a 365 nm wavelength. This property was not observed in the CNPs that were prepared in water (Figure 28) [110]. Hence, the type of liquid used in the PLAL could affect the optical properties of the fabricated nanoparticles.

De-ionized water and isopropanol were used as liquid media in a PLAL experiment to produce CNPs [111]. These liquids were used as they are rich in oxygen, so OH groups can be easily formed. The presence of the OH groups can cause etching on the surface of the CNPs. Using a 1064 nm laser wavelength with a pulse duration of 200 ns, 25 kHz repetition rate, and adjustable power in the range of 0.18–7.52 W, CNPs with different sizes were synthesized. It was found that these parameters assisted in the fabrication of CNPs with size < 100 nm. The best results were associated with a laser power of 4.6 W, which resulted in a more homogeneous size distribution of 75 nm.

An unfocused beam of an Nd:YAG laser of 532 nm wavelength, 10 ns pulse duration, 10 Hz repetition rate, and fluence of 0.131 J/cm^2^ was employed in a PLAL process to irradiate samples consisting of activated carbon (4% ash) in a solution of ethanol and double distilled water [102]. The sample was irradiated for 30 min, which resulted in the synthesis of CNPs of an average size of about 14 nm. When NaOH was added to the solution, while keeping all experimental conditions the same, the CNPs were reduced in size to 4 nm. The photoluminescence properties of the produced CNPs exhibited a spectral peak intensity in the blue region (466 nm) in the absence of NaOH, which was shifted to longer wavelength to the green region (515 nm) when NaOH was included. It was concluded that the liquid media has a significant effect on the size and optical properties of the synthesized CNPs [102].

When different nitrogen-containing precursors dissolved in water were used as liquid media in a PLAL process, the synthesis of nitrogen-doped GQDs (N-GQDs) was successful [103]. Carbon nano-onion (nCNO) powder was pressed into a pellet to form a target material that was immersed in a mixture of water with ammonia, ethylenediamine, or pyridine to form a solution for the PLAL process. The target was irradiated with a 532 nm laser, with a pulse width of 6–8 ns and 50 Hz repetition rate, for 1 h at a fluence of 4.5 J/cm^2^. The obtained N-GQDs had a size of ~14 nm and exhibited PL emission tunability that was dependent on the liquid used. Moreover, these N-GQDs showed the ability to be used as an electrocatalyst [103].

#### 3.2.2. Laser Wavelength

**Figure 29:**
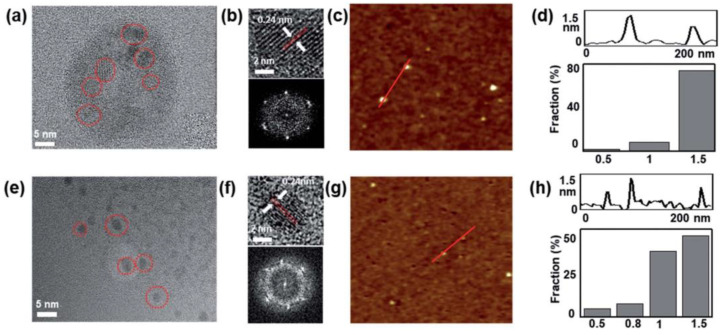
Effect of laser wavelength on the synthesis of GQDs (**a**–**d**) and GOQDs (**e**–**h**). [68].

In order to improve the selectivity production of GQDs and GOQDs, the effect of laser wavelength was investigated [68]. Two wavelengths, 532 nm and 355 nm, were employed in a PLAL process. The different wavelengths were used to irradiate 50 mg of MWCNTs dispersed in 500 mL of ethanol. Regardless of the wavelength used, the size of the produced QDs were in the range of 1–5 nm with thicknesses between 0.5–1.5 nm, indicating a graphene structure of one to a few layers. The main feature of using different wavelengths was the different optical properties observed. This was attributed to the interaction of the laser beam with solvent and colloidal suspension. It was found that the laser wavelength has a strong influence on the decomposition of the solvent used in the PLAL method. Furthermore, laser wavelength can affect the functionalities of the produced QDs. Using a 50 mJ laser pulses with 10 Hz repetition rate, a 532 nm wavelength resulted in the production of GQDs, while a 355 nm wavelength mostly produced GOQDs. The higher photon energy of the 355 nm laser beam resulted in the decomposition of the ethanol, enriching it in OH, causing defects on the surface of the fabricated GQDs. Both factors aided in the transformation of GQDs to GOQDs (Figure 29). This was confirmed from the investigation of the optical properties of the produced QDs from the two wavelengths. Photoluminescence investigation indicated a distinct blue emission of GQDs produced with 532 nm, while GOQDs produced by 355 nm had a blue to green emission [68].

**Figure 30:**
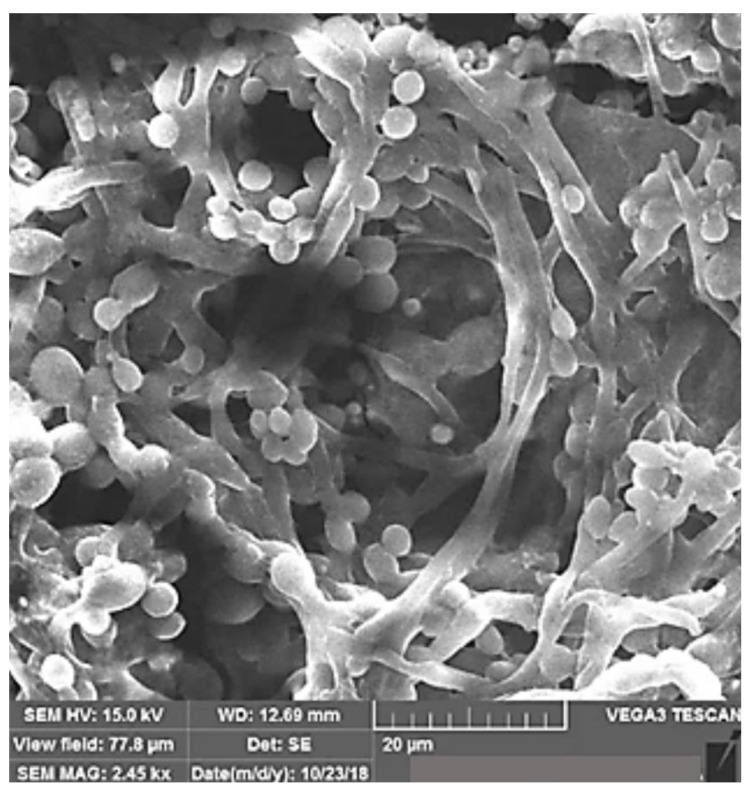
SEM images of graphene-oxide nanostructures using a 532 nm wavelength. [67].

Graphene-oxide nanostructures have also been fabricated using a 1064 nm and 532 nm lasers. The laser irradiated a graphite target immersed in de-ionized water, and graphene-oxide nanoballs and nanowires were produced [67] (Figure 30). Although the experimental conditions were similar to other works that reported the production of graphene nanosheets along with carbon nanostructures, this work [67] did not report the formation of nanosheets. Furthermore, no information on the lens used to focus the laser beam was given, hence, the fluence of the beam was not clear, which is an important factor in determining the final products in a PLAL process.

#### 3.2.3. Laser Fluence

The laser fluence effect on the production of CNPs was also investigated [112]. Spherical CNPs in the range of 4–20 nm were obtained when a 1064 nm laser wavelength with 7 ns pulse length and 15 Hz repetition rate was used in a PLAL experiment. A graphite target was submerged in acetone and irradiated with a laser beam of 0.17, 0.42, 0.70, 0.90, and 1.0 J/cm^2^ fluence for 180 s. It was concluded that changing the laser fluence controlled the size and concentration of the CNPs in the colloidal solution. Moreover, these CNPs showed good tunable photoluminescent properties.

Laser fluence was found to be an important factor for the formation of GQDs when incorporating a femtosecond laser. Irradiating an HOPG target immersed in water for 20 min with a laser beam of 35 fs pulse duration, 800 nm wavelength, 1 kHz repetition rate, and fluence ranging from 20–30 J/cm^2^ resulted in the synthesis of GQDs. When the fluence was reduced below 12 J/cm^2^, no GQDs were formed. This demonstrated that a threshold fluence is required for the production of GQDs [82].

**Figure 31:**
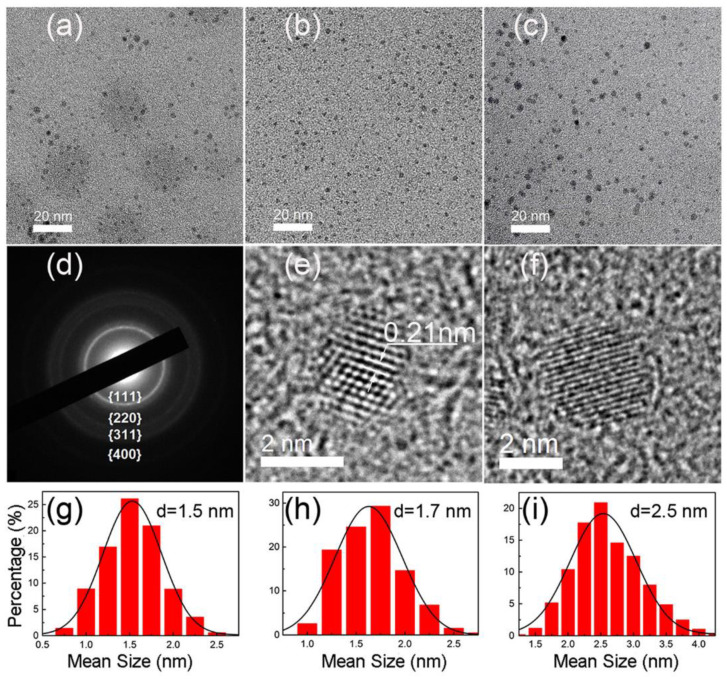
Effect of laser fluence on the production of CDs: (**a**) 150 J/cm^2^, (**b**) 350 J/cm^2^, (**c**) 750 J/cm^2^, (**g**–**i**) size distribution, respectively, (**d**) SAED of c, (**e**) HRTEM of a, (**f**) HRTEM of c. Reprinted with permission from Ref. [104]. 2015, AIP Publishing.

The effect of fluence was also investigated by irradiating carbon powder dispersed in PEG_200N_ [104]. Using an 800 nm wavelength femtosecond Ti:Sapphire laser, the solution was irradiated for 3 h. The fluence was changed between 150 J/cm^2^ to 1000 J/cm^2^, and the resultant CDs were investigated. It was found that as the fluence was increased from 150 J/cm^2^ to 750 J/cm^2^, the size of the CDs increased (Figure 31). However, by increasing the fluence further to 1000 J/cm^2^, the CD size decreased. This was attributed to the increase in temperature and pressure of the plasma plume, which increased the size of the bubble, resulting in larger size CDs. When the fluence was further increased, reaching 1000 J/cm^2^, the ablation efficiency increased significantly, hence, a larger number of CDs were ablated of the already size-reduced CDs [104].

Femtosecond lasers have also been used to fabricate CNPs using the PLAL technique. An 800 nm Ti:sapphire laser was used with 150 fs pulse duration, 1 kHz repetition rate, and a laser power ranging from 100 mW to 400 mW to irradiate 0.1 mg of graphite powder immersed in 50 mL of aminotoluene for 2 h [105]. The produced quantum dots had a mean size of 2.78 ± 0.02 nm with a narrow size distribution when 400 mW of power was used. No results of size dependence on power was reported. However, the effect of increased power on the optical properties of the produced CNPs was performed. It was found, from the FT-IR analysis, that the absorption bands increased with increasing laser power, which in turn affected the photoluminescent properties of the GQDs. The increase in laser power resulted in the CNPs fluorescing with red-shifted wavelengths. This was utilized as a fluorescent nanosensor for pH quantification and monitoring.

#### 3.2.4. Irradiation Time/Pulse Duration/Repetition Rate

Work was done to fabricate CNP with high repetition rate, high fluence, and high number of pulses [113]. Using an Nd:YAG 1064 nm laser with 700 ps pulse length, the experimental parameters were varied to include repetition rates as high as 14 kHz, number of pulses of up to 1800 pulses, and fluence reaching 0.9 J/cm^2^. The colloidal solution was found to contain CNPs and MWCNTs, where the laser fluence was the key parameter that affected the type of produced carbon nanostructures. Increasing the laser fluence favors the formation of CNPs. The size of the CNPs obtained ranged from 42 to 75 nm. The size of the obtained CNPs were much larger than those obtained with lower repetition rate (10 Hz) and longer pulse width (7 ns), which reached an average of 6 nm [101]. This might be attributed to the laser wavelength used and the absorption of the target material to that wavelength.

More investigations on the effect of irradiation time on the size of fabricated CDs was performed [104]. The irradiation time was increased from 2–4 h while irradiating carbon powder dispersed in PEG_200N_ using an 800 nm laser, pulse width 150 fs, and repetition rate of 1 kHz [104]. While keeping the laser fluence fixed at 150 J/cm^2^ with a spot size of 16 μm, the size of the formed CDs was studied. It was observed that with increasing irradiation time, the size of the as-prepared CDs decreased, reaching a size of 1.3 nm after 4 h. Moreover, the size distribution became narrower [104].

Graphene-oxide nanospheres (GONs) were fabricated using PLAL by starting with graphene nanoplatelets dispersed in ethanol [63]. By irradiating the solution with 1064 nm unfocused beam for 60 min, the solution transformed from black to transparent yellow, indicating the production of graphene oxide. After drying at room temperature to remove the ethanol, a brown powder was left that was washed with double-distilled water, sonicated, and centrifuged to extract the graphene-oxide nanospheres, which were then dried at 70 °C for 8 h. The produced GONs had a solid spherical structure with an average size of 137 nm. The spherical shape was attributed to the crumpling of the graphene sheets as a result of the laser irradiation. When the irradiation time was reduced to 30 min and the last step of drying at 70 °C for 8 h was excluded, the produced GONs size reduced to an average of 26 nm [16].

**Figure 32:**
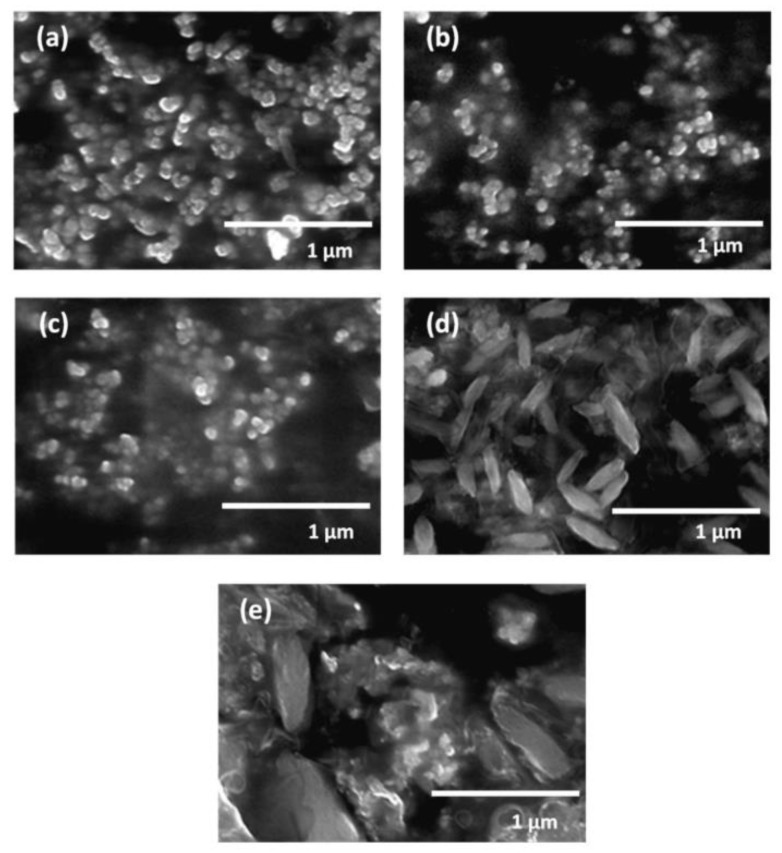
FE-SEM of graphite nanoparticles at laser frequency of (**a**) 1 Hz, (**b**) 10 Hz, (**c**) 20 Hz, (**d**) 50 Hz, and (**e**) 100 Hz. [81].

The effect of laser repetition rate on the size and morphology of the GO nanoparticles after a PLAL process was investigated [81]. A 532 nm Q-Switched laser with pulse width of 1.5 ns was focused on a graphite target submerged in de-ionized water. The maximum available average power of the laser was kept constant at 0.813 mW. The number of laser pulses per second was changed to the values 1, 10, 20, 50, and 100 Hz, which in turn changed the laser fluence to 0.21, 0.025, 0.014, 0.006, 0.008 J/cm^2^, respectively. The change in fluence had a direct effect on the size and morphology of the produced GO nanoparticles, where lower fluence produced spherical particles, while higher fluence gave a more elongated particle (Figure 32). The repetition rate of 10 Hz was considered as the optimum condition, where it gave the highest ablation rate and smallest particle size. Furthermore, when the thermal conductivity of the nanofluids was measured, the fluid with 10 Hz repetition rate gave the highest thermal conductivity.

#### 3.2.5. Target Material

Starting from carbon nano-onions (nCNOs) as a target material immersed in de-ionized water, PLAL was employed to produced GQDs [92]. A 532 nm laser was used with a pulse width of 5–7 ns and repetition rate of 10 Hz. The laser power was set at 1.3 W and left to irradiate the sample for 7 h to ensure that a sufficient number of GQDs was produced. The average diameter of the fabricated GQDs was about 1.8 nm with a thickness of nearly single-layer graphene. The produced GQDs also showed photoluminescence properties with emission peaking around 445 nm. It was concluded that laser parameters such as power, pulse width, and focusing spot size can affect the size distribution and photoluminescence properties of the produced GQDs.

GOQDs were fabricated through PLAL starting from coal as a carbon source [95]. One gram of coal was dispersed in 30 mL of ethanol and irradiated by a laser beam of 355 nm wavelength, 10 ns pulse width, 10 Hz repetition rate, and 0.1 J of laser energy. An irradiation time of 5 min succeeded in downsizing the bulk coal particles to GOQDs of a few nanometers. The diameter of the produced GOQDs ranged from 5 to 30 nm with thickness of one-to-few-layer graphene sheets. The yield from this PLAL process was calculated and found to be 18%. The produced GOQDs exhibited good optical properties such as brightness in emitting green light and photostability even after a period of two months. This makes the GOQDs promising for biomedical applications, especially since they possess low toxicity.

**Figure 33:**
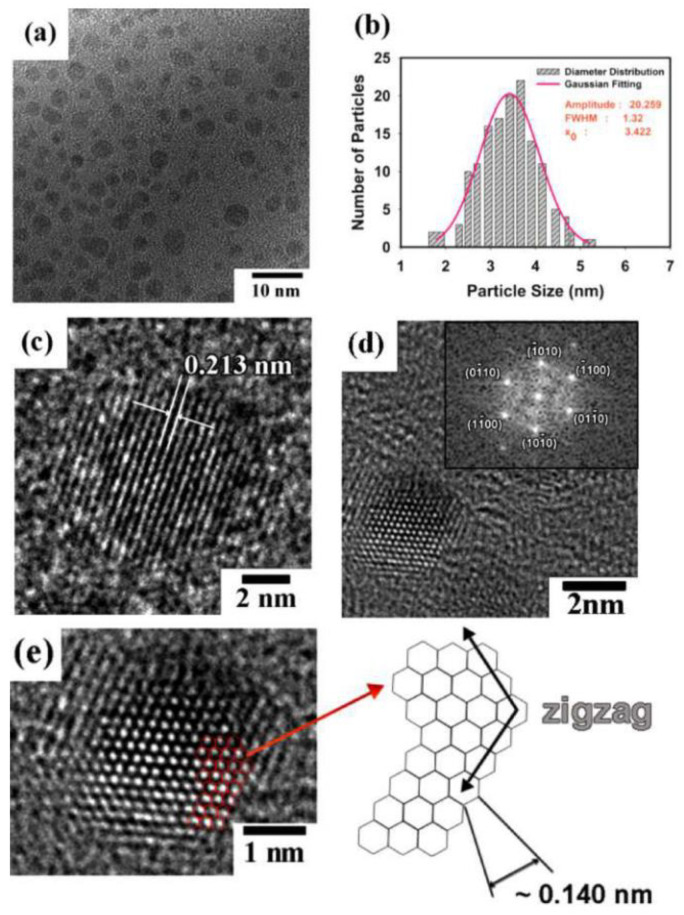
(**a**) HRTEM of GQDs, (**b**) size distribution, (**c**) lattice spacing, (**d**) FFT analysis, (**e**) zigzag structure of the particles’ edges. Reprinted with permission from Ref. [114]. 2013, Elsevier.

Starting with a mixture of nickel (II) oxide powder in benzene as a carbon source, a laser beam of wavelength 1064 nm irradiated the solution for 30 min. The laser beam had 10 ns pulse duration, 10 Hz repetition rate, and laser energy of 30 mJ/pulse. After irradiation, the solution was centrifuged to obtain the GQDs in the supernatant. The benzene was separated from the GQDs through rotor evaporation, and then the GQDs were dissolved in de-ionized water. The size of the fabricated GQDs were in the range of 2–6 nm with a very narrow size distribution (Figure 33). Moreover, the GQDs showed strong fluorescence in the visible region and displayed suitability as a biomarker [114].

#### 3.2.6. Extending PLAL Technique

**Figure 34:**
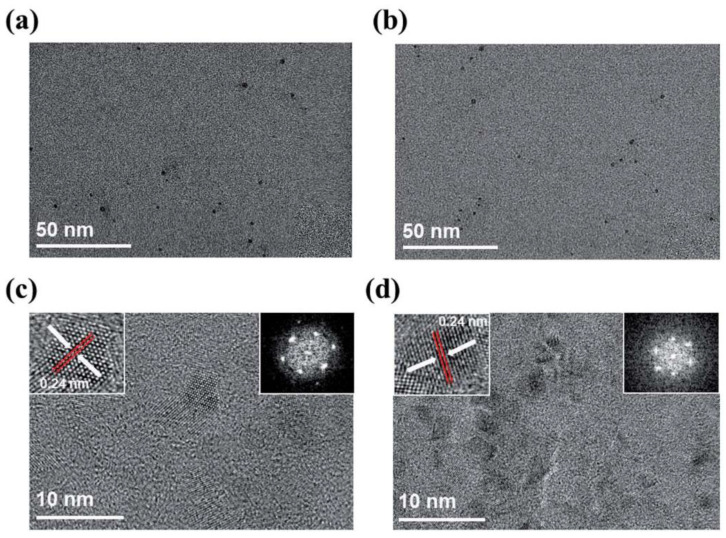
(**a**) HRTEM images of (**a**,**c**) on-GQDs and (**b**,**d**) off-GOQDs [96].

PLAL was also used with the assistance of high-power sonication [96]. Starting with 500 mg of graphite flakes emersed in 200 mL of ethanol, a 355 nm laser with 10 Hz repetition rate and 10 nm pulse width was used to irradiate the sample. The laser power was set at 1.5 W and the irradiation time was 30 min. The size of the produced GQDs, with or without sonication, was in the range of 3–4 nm. However, it was found that without sonication, the fabricated particles had oxygen-rich functional groups on the surface of the particles. Hence, GOQDs were formed when no sonication was employed. This was not the case when high-power sonication assisted the PLAL process and GQDs were produced (Figure 34). As a result, there were different optical properties of the produced GQDs when sonication was turned on or off. Without sonication, the GOQDs exhibited blue and green mixed emission, while with sonication, the GQDs had a distinct blue emission. The effect of sonication during PLAL shed light on the mechanism of GQD formation. When no sonication was used, the nanoparticle formation mechanism was mainly the bottom-up approach of laser ablation in liquids (LAL); however, when sonication was incorporated, the formation mechanism was mostly a top-down approach through laser fragmentation in liquid (LFL) [96].

Doping GQDs with nitrogen (N-GQDs) improved their optical properties, resulting in broadening of their emission band. Furthermore, nitrogen doping enabled the tuning of the N-GQDs luminescence [18]. DMF was used as a nitrogen source solvent, submerging a graphite target in a PLAL process. A 532 nm laser wavelength with 10 ns pulse width, 100 Hz repetition rate, and 7.5 J/cm^2^ fluence irradiated the graphite target. The PLAL processed was followed with a solvothermal treatment where the colloidal dispersion was placed in a sealed glass bottle and heated in an oven at 65, 90, and 120 °C for 72 h. As a result, the PLAL produced GQDs that were doped with nitrogen (N-GQDs). The subsequent solvothermal treatment of the N-GQDs assisted in the reduction of oxygen functional groups and the increase of the nitrogen content of the N-GQDs. At 120 °C, the N-GQDs exhibited the highest photoluminescence intensity, making the product a good candidate for an optical sensor [18].

Nitrogen-doped GQD production has also been attempted with a focus on the effect of sonication during the PLAL process on the doping process [97]. Using a 1 W laser beam with wavelength 355 nm, 10 Hz repetition rate, and 10 nm pulse width, natural graphite flakes immersed in ethanol and diethylenetriamine (DETA) were irradiated for 30 min with sonication off and on. The produced N-GQDs were dried overnight at 80 °C, after which characterization took place. The size of the N-GQDs, in both cases of sonication off and on, had an average size of less than 6 nm. However, the optical properties of the two cases were different. Contrary to expectation, it was found that N-GQDs that were produced without sonication had better PL properties. This was attributed to the reduction in the exposure time of the graphite flakes to the laser ablation due to the vigorous sonication. It was concluded that the PLAL process without sonication was more effective in incorporating nitrogen atoms to the GQDs [97].

Another method for the production of N-doped GQDs incorporated laser-induced-graphene (LIG) as a carbon precursor in a PLAL process [106]. The LIG dispersed in a solution showed more advantages as a precursor when compared to using a solid graphite target. Such a method helped in obtaining smaller uniform particle size with good dispersion. Starting with a polyiminde (PI) film, irradiated with a 10.6 μm and 3.5 W CO_2_ laser, the LIG was obtained. The LIG film was then scrapped to obtain LIG powder, which was mixed with water and ammonia to prepare a suspension. After ultrasonic treatment, a PLAL process was performed where the suspension was irradiated with a 1030 nm femtosecond laser with 100 kHz repetition rate, 8 W power, and 365 fs pulse duration. The colloidal solution had a light brown color, indicating the presence of GQDs. When comparing the produced GQDs and the N-doped GQDs, the N-doped GQDs had a smaller size, in the range of 3 nm, and were more uniform, with 1–3 layers thickness. The PL spectrum of the N-doped GQDs showed higher intensity when compared to undoped GQDs, while fluorescing in the blue-green region [106].

Post-treatment of the PLAL process for the production of CNPs was investigated [40]. Starting with commercially available charcoal powder submerged in ethanol, an unfocused laser beam of 1064 nm wavelength, 10 ns pulse width, 10 Hz repetition rate, and 750 mJ pulse energy was used to irradiate the sample for 20 min. The solution was then left to dry at room temperature for 48 h. Double-distilled water was then added to the produced particles, and the colloidal solution was sonicated and then centrifuged to obtain water-soluble CNPs (W-CNPs). The post-treatment of the CNPs was found to result in well-dispersed particles with an average particle size of 21 nm. The W-CNPs illustrated excitation-dependent PL emission with intense emission in the blue region (445 nm) when excited with 380 nm wavelength. Furthermore, W-CNPs exhibited cytocompatibility and photostability after in vitro tests on human cardiomyocytes [40].

To improve the production of luminescent carbon nanoparticles, a two-stage PLAL process was suggested [115]. In the first stage, an unfocused laser beam of 1064 nm wavelength was made to fall on a graphite target submerged in a liquid. This beam offered a low fluence of 2 J/cm^2^ to ablate the graphite target. In the second stage, the graphite target was removed and the colloidal solution was irradiated by a focused beam offering a high fluence, reaching 15 J/cm^2^, to shred the particles formed in the first stage. Splitting the process into two stages helped meet the conflicting requirements, in terms of fluence, for the ablation and refinement of the fabricated particles. Incorporating urea in stage one or two or both helped in improving the luminescent properties of the CNPs [115].

**Figure 35:**
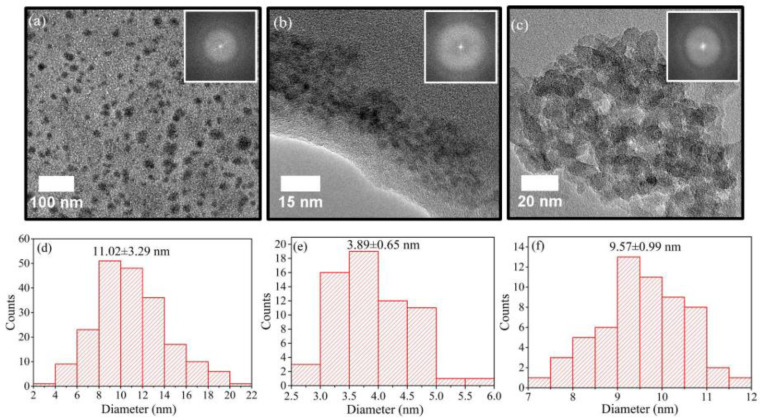
TEM images of soybean-derived NPs: (**a**) HTC-CDs, (**b**) annealed-CDs, (**c**) LA-CDs, (**d**–**f**) the size distributions, respectively. [118].

PLAL was combined with other methods to fabricate carbon nanoparticles. Starting with ground soybean residuals, successive processes of hydrothermal carbonization (HTC), annealing at high temperature, and laser ablation (LA) in NH_4_OH solution resulted in the formation of carbon nanoparticles (Figure 35). The laser ablation was performed using a 532 nm laser with 50 Hz repetition rate and 2 ns pulse duration, which assisted in the introduction of nitrogen-surface functional groups that affected the photoluminescent features of the nanoparticles. Hence, this method provided a simple and green method to produce stable photoluminescent carbon nanoparticles with enriched nitrogen-surface functional groups from biowaste [118].

## 4. Applications of PLAL Graphene Nanostructures

Graphene-based materials have gained great interest since they have features that enable the tunability of their physical, electronic, and mechanical properties [69,119]. For example, the electronic conductivity and catalytic properties of carbon structures are affected greatly by their crystallinity. There are many methods reported for the fabrication of graphene and its derived materials by incorporating lasers other than PLAL method [120,121,122,123,124,125,126,127]. Fabrication applications include the fabrication of electrodes for batteries [128,129], supercapacitors [130,131,132], resistive switching memory devices [133], micro-capacitors [134,135], paper-based liquid sensors [136], super-hydrophilic porous membranes [137,138], and 1D fiber electronics [139]. Moreover, graphene and lasers were used for signal enhancement for laser-induced breakdown spectroscopy [140]. Although synthesis of nanostructures via PLAL is heavily investigated, there is still a lack of investigation in the practical applications of its produced nanostructures. Since this review is specifically for the PLAL technique, the applications that are highlighted below are specific to this method.

### 4.1. Bio-Applications

Imaging in the medical field is of high importance. The ability to label and track living cells will assist in better diagnose of diseases. Semiconductor quantum dots, such as CdSe, HgTe, InAs, and HgCd are good candidates as they fluoresce efficiently and do not photobleach, giving them long stability. However, these semiconductor quantum dots are very toxic to cells, rendering them unsuitable in biomedical applications. GQDs have emerged as a biocompatible material for medical applications and have shown properties of stable fluorescence, making them very suitable for medical imaging [64,71,141,142]. Since PLAL can be used to fabricate nanoparticles in water, for example, this technique can be an efficient method for the production of safe nanostructures for medical applications.

**Figure 36:**
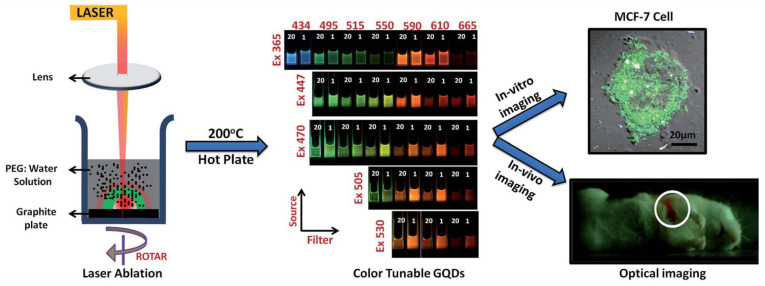
GQDs exhibiting color-tunable emission. [109].

GQDs were fabricated using PLAL for the biomedical application of cell tracking and single-cell imaging [109]. The produced GQDs were less than 10 nm in size. By refluxing the GQDs at 200 °C for 20 min and 1 h, the GQDs exhibited an excitation-independent broad emission peaking at 600 nm and excitation-dependent color-tunable emission, respectively (Figure 36). The optical properties of the GQDs were found to be controlled by their oxidation levels and could fluoresce for up to 48 h post-incubation during in vivo imaging. Hence, the synthesized GQDs proved to be suitable as fluorescent biomarkers [109].

An assessment of GOQDs for a bioimaging application has been undertaken [95]. By performing cytotoxicity tests, the GOQDs showed that they are highly biocompatible with low cytotoxicity level. Therefore, GOQDs can be used as a PL probe in the bioimaging field. This was further confirmed when GONs were used on human vascular smooth muscles cells in an in vitro examination [16]. The GONs were found to internalize the cells within 6 h, while the photoluminescence emission was not affected, indicating their photostability. Moreover, they demonstrated no cytotoxic effect on the cells. Hence, GONs synthesized by PLAL were shown to be suitable as fluorescent non-toxic nanoparticles for biomedical applications.

Ascorbic acid (AA) plays an important role in the human body, being involved in processes such as iron absorption and infection protection. Therefore, monitoring AA is of significant importance. Nitrogen-doped GQDs (N-GQDs) were found to have photoluminescence properties that were quenched in the presence of Fe^3+^ [18]. Hence, N-GQDs showed excellent sensing properties as a “turn off” detector for Fe^3+^. The system of N-GQDs/Fe^3+^ was then tested as a sensor for AA and showed excellent properties as an “on-off-on” detector [18]. A similar application was examined with N-GQDs prepared by a two-step process [106]. Starting with the fabrication LIG and using it as a precursor in a PLAL process in the presence of ammonia, N-GQDs were synthesized that exhibited PL properties that were quenched in the presence of Fe^3+^. Such work opens the door for more biocompatible fluorescence sensors.

GQDs were obtained from irradiating a mixture of nickel (II) oxide powder in benzene, as a carbon source, by a 1064 nm laser for 30 min. The GQDs showed strong fluorescence, reaching a maximum PL emission intensity of 450 nm when excited by a 350 nm wavelength. When bacteria were exposed to the fabricated GQDs and incubated for 3 h, confocal microscopy images showed an enhanced imageability of the bacteria under excitation wavelengths of 405, 488, and 561 nm with high contrast. Since the GQDs were reported to be non-toxic, this makes them highly suitable for biological applications [114].

N-doped micropore carbon quantum dots (NM-CQDs) were synthesized using PLAL utilizing *Platanus* biomass as a carbon precursor. The NM-CQDs exhibited high dual wavelength (indigo–blue) photoluminescence emission. Moreover, the NM-CQDs had a stable emission in various conditions such as different temperatures, pH levels, salt ionic concentration, and excitation wavelengths. These features rendered the NM-CQDs suitable for bioimaging applications since they also exhibited good internalization in different cells [116].

Tunable broadband fluorescence CDs have been fabricated via PLAL by irradiating carbon powder in PEG_200N_ using a 1064 nm laser wavelength, 8 ns pulse width, 20 Hz repetition rate, and 100 mJ pulse energy [117]. The average size of the produced CDs was approximately 3 nm. The tunable broadband fluorescence of the synthesized CDs when excited with a 390 nm wavelength was attributed to different factors such as functionalization of the CDs and the inhomogeneous particle size distribution, yielding different quantum-confining states.

### 4.2. Catalysis

An investigation of the effectiveness of GO nanosheets when incorporated with metallic NPs for photodegradation was conducted [14]. Starting with a solution containing GO nanosheets dispersed in de-ionized water, Ag and Cu targets were submerged in the solution to conduct a PLAL procedure. An Nd:YAG laser with 7 ns pulse duration, 10 Hz repetition rate, and 3.6 W average power was used to irradiate the Ag and Cu targets that were submerged in the GO solution. Spherical AgNPs@GO and CuONPs@GO were fabricated with sizes in the ranges 3.5–27.3 nm and 5.3–13.6 nm, respectively. These NPs were tested for the degradation of methylene blue (MB) under visible light. Results showed that although GO alone had a degradation efficiency of 89.9%, by incorporating AgNPs, the efficiency increased to 91.7%, reaching 93.1% with CuONPs in a 40 min time interval. Moreover, the AgNPs@GO and CuONPs@GO maintained performance after five cycles, indicating their reuse feasibility.

PLAL was also used to fabricate tungsten-oxide/reduced graphene-oxide (WO_3_-rGO) composites for photocatalytic water decontamination [98]. A mixture of WO_3_ NPs and monolayers of graphene oxide dispersed in de-ionized water was irradiated by a 355 nm laser beam at 280 mJ/pulse energy for 30 min. During the irradiation, the photo-induced electrons in the WO_3_ reduced GO to rGO and assisted in anchoring the WO_3_ NPs on the graphene sheets, forming WO_3_–rGO composite. When the WO_3_–rGO composite was tested for photodegradation of MB in water, it showed an enhancement in efficiency when compared to pure WO_3_ NPs.

### 4.3. Energy-Relevant Applications

Fabrication of transparent conductive graphene films using a PLAL technique has been performed [77]. The suspension obtained from PLAL underwent vacuum filtration through a mixed cellulose ester (MCE) membrane. The graphene was then pressed firmly on a glass substrate to remove any bubbles between the membrane and the substrate. The membrane was later dissolved by immersion in acetone. By annealing the graphene film at 250 °C for 2 h, the conductivity of the film reached 1000 S/m and its transparency was around 75% at 550nm.

The fabrication of platinum-nanoparticle–graphene hybrids was performed using PLAL to produce cathode catalysts for Li–air batteries [99]. This was achieved by preparing an aqueous graphene solution in a glass container and fixing a platinum plate on its side. A 355 nm laser with 10 Hz repetition rate, 5 ns pulse duration, and 2 J/cm^2^ fluence was used to irradiate the platinum plate for a duration of 30 min. This resulted in the formation of 3–5 nm Pt NPs onto the graphene nanosheet GNS. The PtNP–GNS hybrids exhibited good performance as cathode catalysts in Li–air batteries, with a discharge capacity of 4820 mA h/g.

Incorporating femtosecond lasers in the PLAL technique has assisted in the production of QDs, which were utilized in the fabrication of deep UV (DUV) photodetectors. C-doped ZnO QDs have been fabricated by irradiating a Zn_2_N_3_ target immersed in ethanol [17] using an 800 nm Ti-sapphire laser with 150 fs pulse duration and 76 MHz repetition rate. The produced C-doped ZnO QDs were in the size range of 3.4 nm. Using the colloidal solution as an active layer resulted in a development of a DUV photodetector that operateed in the UV–C region and had high responsivity, fast response, and stable switching performance.

PLAL was used to manufacture MWCNTs/Si photodetectors [12]. The MWCNTs produced by the PLAL process were affected by the wavelength incorporated in the process. Two colloidal solutions of MWCNTs were prepared using 532 nm and 1064 nm laser wavelengths. The colloidal solutions were deposited on silicon substrate to form MWCNTs/Si photodetectors. The effect of the wavelength used in the PLAL on the performance of the photodetector was investigated. It was concluded that the MWCNTs/Si photodetectors that were prepared with the 532 nm laser had better responsivity and detectivity, compared to using a 1064 nm wavelength, reaching a maximum responsivity of 0.53 A/W and detectivity of 2.1 × 10^12^ Jones.

Furthermore, a WO_3_–rGO composite was prepared by PLAL using a 355 nm laser wavelength, which irradiated a mixture of WO_3_ NPs and monolayers of graphene oxide dispersed in de-ionized water. This composite was examined as an electrode material for supercapacitor application and exhibited an enhanced performance of capacitance when compared with pure WO_3_ NPs. Hence, such a composite may prove beneficial for energy storage applications [98].

## 5. Summary and Outlook

Graphene and its derivatives have found their way into many industrial applications sectors, such as optoelectronics, energy devices, and bioimaging, to name a few. Hence, the development of large-scale fabrication methods to synthesize high-quality graphene nanosheets and graphene quantum dots is highly desirable. Although numerous methods have been established, many entail a high cost. One promising method to reduce the cost of graphene nanostructures production is pulsed laser ablation in liquid (PLAL) which has been shown to be single-step, easy, fast, and relatively inexpensive.

However, a successful controlled production would require the control of the experimental parameters that are related to all aspects of the process. For example, the laser source used for the ablation entails parameters such as the wavelength of the laser beam, its energy, pulse width, repetition rate, and focusing optics. Furthermore, the liquid medium chosen in the process has great effect on the structure, morphology, and optical properties of the synthesized nanostructures. Moreover, the target which is used as a carbon precursor is also of high importance. All these parameters are interlinked and collectively influence the laser–matter interaction, which form the bases for the fabrication mechanism.

In this review, recent developments in the utilization of PLAL techniques for the synthesis of graphene nanosheets and graphene quantum dots have been surveyed. This survey is intended to provide a useful guide for researchers and offer a motivation to invest more work into correlating experimental results with theory. A better understanding of the physical and chemical processes involved in graphene synthesis via PLAL will provide a better control protocol for fabricating final products with specific features.

This review discusses the importance of graphene as a product and the advantages of PLAL as a promising technique for its production. The fundamental mechanisms of laser ablation in liquids are summarized, and the influence of experimental parameters on this mechanism is explained. The variety of experimental setups in a PLAL process is also illustrated, along with major characterization methods used to identify produced nanostructures, such as XRD, XPS, and Raman spectroscopy.

References covered in this review were largely published in the last five years. They offer diverse experimental conditions for target materials, liquids, and laser parameters. Moreover, some extend the PLAL by integrating it with other techniques to better control the produced nanostructures. Others have investigated the use of biowaste to synthesize graphene in an effort to reduce waste and transform it into useful graphene. All these experimental works offer a pool of information that is necessary to understand the dynamics of the PLAL process in pursuit of a more controllable mechanism.

PLAL has exhibited great advancements as a synthesis technique for graphene nanostructures; however, many challenges need to be overcome for it to compete as an industrial production process. Of these challenges, the low ablation rate, which in turn translates to relatively low concentrations of the fabricated particles, hinders scaling-up. Furthermore, although much research has been devoted to synthesizing graphene and its derivatives via PLAL processes, work on their potential applications is still limited. For example, GQDs and GOQDs have demonstrated excellent photo-luminance properties that suit them for bioimaging applications. To better control the products of a PLAL process and tune product properties, more understanding of fundamental process mechanisms is required. This requires more theoretical and computational work to be correlated with experimental results. One of the advantages of graphene production via PLAL is the reduction of contamination, as it is formed in a colloidal solution, guaranteeing its purification. Attempts to deposit graphene films onto substrates through a PLAL process replace the need to transfer the graphene sheets onto substrates and, in turn, reduce the possibility of degrading the graphene during the transfer step.

We hope that this review aids in the understanding of PLAL techniques as a tool for the production of graphene nanosheets and graphene quantum dots and that it may serve as a reference for the further development of PLAL as a synthesis technique that can be utilized in diverse applications.

## Figures and Tables

**Table 1:** Graphene nanostructures analysis techniques and its main features.

Characterization Method	Description		Reference
XRD	Graphene	2θ = 26.5° (002), 42.3° (100), 44.5°(101), 54.6° (004), and 77.4° (110)	[63]
	Graphene oxide	2θ ≅ 9–11° (001), shifts depending on amount of trapped oxygen to 2θ ≅ 12–17°	[16,24]
XPS	C 1s O 1s	Binding energy = 284 eV Binding energy = 532 eV	[64]
Raman spectroscopy	D-band G-band 2D-band	Raman shift = 1330–1360 cm^−1^ Raman shift = 1560–1600 cm^−1^ Raman shift = 2400–1700 cm^−1^ *I_2D_ > I_G_* decreased layer thickness *I_D_ > I_G_* increased disorder and poor quality	[65,66]
UV-Vis spectroscopy	Graphene Carbon material	No peak due to transparency Peak in the range 260–270 nm (π–π* transition of C=C bond). Peak in the range 270–350 nm (n-π* transition of the C=O bond).	[63,67]
PL emission spectroscopy	GQDs GOQDs	Blue emission Mixed blue and green emission	[68]
FTIR spectroscopy	O-H stretching C-H stretching 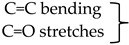 C-O-C bonds	3420 cm^−1^ 2930 cm^−1^ 1633 cm^−1^ 1068 cm^−1^	[69]

**Table 2:** Experimental conditions for the fabrication of graphene and graphene-oxide nanosheets via PLAL technique.

Wavelength	Fluence/Energy/Power	Repetition Rate	Pulse Width	Target/Solvent	Irradiation Time	Size	Ref.
248 nm	2.5, 3.12 to 3.75 J/cm^2^	10 Hz		Graphite target/ de-ionized water	30 min	Bilayer graphene	[66]
532 nm	5J/cm^2^	10 Hz	5 ns	Graphite target/ distilled water with assisted electric field	Few min^–2^ h	2–6 layers flower-like	[54]
532 nm	0.5, 2.2 and 3.6 J/cm^2^	10 Hz	5 ns	Graphite disk/ distilled water	15 min	1–4 layers	[55]
532 nm	0.5, 0.8, 1.1, 1.4, and 1.8 J/cm^2^	5 Hz	7 ns	Graphite plate/ liquid nitrogen	--	Bi- to multi-layer	[56]
532 nm	0.5 J/mm^2^	--	7 ns	Graphite plate/ acetone	10, 20, 30, 40, 60 s	Bi- and multi-layer graphene	[58]
532 nm	0.5 J/cm^2^	5 Hz	7 ns	Graphite target/ Liquid nitrogen, distilled water	--	few-layer graphene	[65]
532 nm	0.4 J/cm^2^	10 Hz	6 ns	Graphite rod/ distilled water	10 min	rGO nanosheets	[76]
532 nm	1 J/cm^2^	5 Hz	7 ns	HOPG/ NMP, SDBS	2 h	thickness 1 nm and 10 μm in size.	[77]
532 nm	0.7 J/cm^2^	5 Hz	7 ns	Graphite target/ liquid nitrogen, de-ionized water, 0.01 M CTAB	5000 pulses	Few-layer GO	[78]
532 nm	0.5 J/cm^2^	5 Hz	7 ns	Graphite targets/ 0.02, 0.04, 0.06, 0.08, and 0.1 M CTAB	--	multilayer GO nanosheets	[79]
532 nm	0.5 J/ cm^2^	5 Hz	7 ns	Graphite targets/ liquid nitrogen and 0.04 M CTAB	5000 pulses	Multilayer GO nanosheets	[80]
532 nm	0.813 mW	1, 10, 20, 50, and 100 Hz	1.5 ns	Graphite sheet/ de-ionized water	10 min	110–287 nm	[81]
800 nm	20–30 J/cm^2^	1 kHz	35 fs	HOPG/ water	20 min	PG 6 layers thick Pores 15–20 nm	[82]
1064 nm	0.92 J/cm^2^	10 Hz	10 ns	Graphene nanoplatelets/ ethanol	30 min	26 nm	[16]
1064 nm	0.1, 0.2, 0.3, and 0.4 J.	10 Hz	10 ns	Graphite flakes/ de-ionized water	--	3–10 layers	[32]
1064 nm	0.6 J/cm^2^	5 Hz	7 ns	Graphite target/ water @ Temp: 0, 20, 35, 50 and 65 °C	1000 s	Few-layer graphene	[57]
1064 nm	1.5 J/cm^2^	5 Hz	7 ns	Graphite plate/ 0.02, 0.04, 0.06, 0.08, and 0.1 M CTAB	8000 pulses	few layers graphene	[62]
1064 nm	600 mJ	10 Hz	10 ns	Graphene nanoplatelets/ ethanol	60 min	100–300 nm	[63]
1064 nm	6 J/cm^2^	5 Hz	10 ns	Graphite target/ Liquid nitrogen	20 min	few-layer graphene	[83]
1064 nm	1.5 J/cm^2^	5 Hz	7 ns	Graphite plate/ distilled water, acetone, alcohol, CTAB	1000 s	Few-layer graphene	[84]
1064 nm	1.5 J/cm^2^	5 Hz	7 ns	Graphite plate/ distilled water, liquid nitrogen, acetone, alcohol, 0.01M and 0.1M CTAB	1000 s	Few-layer graphene	[85]
1064 nm	0.5, 0.8, 1.1, 1.4 and 1.8 J/cm^2^	5 Hz	7 ns	Graphite plate/ Liquid nitrogen	5000 pulses	few-layer graphene	[86]
1064 nm	80 mJ 160 mJ	--	7 ns	Graphite target/ water	100 pulses	MWCNTs diameter 25–75 nm PG sheet pours 7–16 nm	[87]
1064 nm	60 J/cm^2^	10 Hz	10 ns	dry-cell graphite electrode (DGE)	20–50 min	Bilayer graphene	[88]
532 nm 1064 nm	15.4 J/cm^2^	2 Hz	9 ns	Graphite pellet/ double-distilled water	30 min	MWCNT diameter 20–75 nm	[12]
532 nm 1064 nm	50 mJ/pulse	5 Hz	10 ns	Flexible graphite targets and nuclear graphite/ acetone, DMF, de-ionized water	5 min	<10 layers	[89]
532 nm 1064 nm	0.5 J/cm^2^ 0.8 J/cm^2^	5 Hz	7 ns	Graphite plate/ liquid nitrogen	5000 pulses	Bilayer graphene	[90]
266 nm 532 nm 1064 nm	0.38 J/cm^2^ (@266 nm) 1.33 J/cm^2^ (@532 nm) 3.33 & 6.61 J/cm^2^ (@1064 nm)	10 Hz	18 ns	Graphite target/ double-distilled water.	--	multilayer rGO nanosheets	[91]

**Table 3:** Experimental conditions for the fabrication of GQDs and GOQDs via PLAL technique.

Wavelength	Fluence/Energy/Power	Repetition Rate	Pulse Width	Target/Solvent	Irradiation Time	Size	Ref.
355 nm	0.50, 0.75, 1.00, 1.50, and 2.00 J	10 Hz	--	MWCNTs/ ethanol	10 min	1–5 nm 3–4 layers	[38]
355 nm	0.1 J	20 Hz	10 ns	Coal/ ethanol	5 min	5–30 nm	[95]
355 nm	1.5 W	10 Hz	10 ns	graphite flakes/ ethanol	30 min	on-GQDs ~3.8 nm off-GOQDs ~4.1 nm	[96]
355 nm	1 W	10 Hz	10 ns	Graphite flakes/ ethanol with DETA	30 min	<6 nm	[97]
355 nm	280 mJ/pulse	--	--	WO_3_ NPS/ de-ionized water	30 min	--	[98]
355 nm	2 J/cm^2^	10 Hz	5 ns	Aqueous graphene dispersion	30 min	Thickness 1 nm	[99]
532 nm	7.5 J/cm^2^	100 Hz	10 ns	Graphite powder/ DMF	--	1.5–7.5 nm	[18]
532 nm	1.30, 0.11, 0.57, 0.95 W	10 Hz	5–7 ns	nCNOs pellet/ de-ionized water	7 h	1.8 nm diameter Single-layer	[92]
532 nm	0.8 J/cm^2^	10 Hz	7 ns	Glassy carbon plate/ de-ionized water	5 min	10–20 nm	[100]
532 nm	1 J/cm^2^	10 Hz	7 ns	Glassy carbon plate/ de-ionized water and THF	30 min	6–15 nm	[101]
532 nm	0.131 J/cm^2^	10 Hz	10 ns	activated carbon (4% ash)/ ethanol + double-distilled water ± NaOH	30 min	4–14 nm	[102]
532 nm	4.5 J/cm2	50 Hz	6–8 ns	nCNOs pellet/ ammonia, ethylenediamine, pyridine	1 h	14 nm	[103]
532 nm	0.21, 0.025, 0.014, 0.006, 0.008 J/cm^2^	1, 10, 20, 50, 100 Hz	1.5 ns	Graphite target/ de-ionized water	10 min	110–287 nm	[81]
800 nm	--	76 MHz	150 fs	Zn_3_N_2_ target/ ethanol	--	~ 4 nm	[17]
800 nm	20–30 J/cm^2^	1 kHz	35 fs	HOPG/ water	20 min	S 2–5 nm	[82]
800 nm	150–1000 J/cm^2^	1 kHz	150 fs	carbon powder/ PEG_200N_	3 h	1–4 nm	[104]
800 nm	100–400 mW	1 kHz	150 fs	graphite powder/ aminotoluene liquid	2 h	2.87 nm	[105]
1030 nm	3.5 W	100 kHz	365 fs	LIG/ water and ammonia	--	3 nm 1–3 layers	[106]
1064 nm	3.6 W	10 Hz	7 ns	Silver/copper disk/ GO + de-ionized water	--	3.5–27.3 nm	[14]
1064 nm	750 mJ/pulse	10 Hz	10 ns	Charcoal powder/ ethanol	20 min	~21 nm	[40]
1064 nm	3.0 J/pulse	2 Hz	5 ms	Graphite target/ ethanol	Stage 1: 5000 pulse Stage 2: 25,000	200–500 nm	[107]
1064 nm	3.0 J/pulse	2 Hz	5 ms	Graphite target/ DMF	Stage 1: 5000 pulse Stage 2: 25,000	80–130 nm	[108]
1064 nm	40 mJ	10 Hz	6 ns	Graphite plate/ PEG–water solution	30 min	<10 nm	[109]
1064 nm	6.0 × 10^6^ W/cm^2^	--	--	Graphite powder/ water PEG_200N_	2 h	~3 nm	[110]
1064 nm	0.18–7.52W	25 kHz	200 ns	Graphite target/ de-ionized water, isopropanol	--	<100 nm	[111]
1064 nm	0.17, 0.42, 0.70, 0.90, 1.0 J/cm^2^	15 Hz	7 ns	Graphite disk/ acetone	180 s	4–20 nm	[112]
1064 nm	0.3, 0.6, 0.9 J/cm^2^	10, 12, 14 kHz	700 ps	Graphite rods/ de-ionized water	1200, 1500, and 1800 pulses	42–75 nm	[113]
1064 nm	30 mJ/pulse	10 Hz	10 ns	A mixture of nickel (II) oxide powder/benzene	30 min	2–6 nm	[114]
1064 nm	Stage 1: 2 J/cm^2^ Stage 2: 15 J/cm^2^	10 Hz	10 ns	Graphite target/ urea ± DIW	Stage 1: 15 min Stage 2: 60 min	<100 nm	[115]
1064 nm	20 mJ/pulse	10 Hz	3–6 ns	waste *Platanus* biomass/ formamide	30 min	8 nm	[116]
1064 nm	100 mJ	20 Hz	8 ns	carbon powder/ PEG_200N_	--	3 nm	[117]
355 nm 532 nm	50 mJ	10 Hz		MWCNTs/ ethanol	10 min	1–5 nm	[68]
532 nm 1064 nm	80 mJ	10 Hz 20 Hz	9 ns	graphite plate/ de-ionized water	45 min	nanoballs 2.68 µm nanowires 1.89 µm	[67]
